# Stochastic shielding and edge importance for Markov chains with timescale separation

**DOI:** 10.1371/journal.pcbi.1006206

**Published:** 2018-06-18

**Authors:** Deena R. Schmidt, Roberto F. Galán, Peter J. Thomas

**Affiliations:** 1 Department of Mathematics and Statistics, University of Nevada, Reno, Reno, Nevada, United States of America; 2 Department of Electrical Engineering and Computer Science, Case Western Reserve University, Cleveland, Ohio, United States of America; 3 Department of Mathematics, Applied Mathematics and Statistics, Case Western Reserve University, Cleveland, Ohio, United States of America; Rush University Medical Center, UNITED STATES

## Abstract

Nerve cells produce electrical impulses (“spikes”) through the coordinated opening and closing of ion channels. Markov processes with voltage-dependent transition rates capture the stochasticity of spike generation at the cost of complex, time-consuming simulations. Schmandt and Galán introduced a novel method, based on the stochastic shielding approximation, as a fast, accurate method for generating approximate sample paths with excellent first and second moment agreement to exact stochastic simulations. We previously analyzed the mathematical basis for the method’s remarkable accuracy, and showed that for models with a Gaussian noise approximation, the stationary variance of the occupancy at each vertex in the ion channel state graph could be written as a sum of distinct contributions from each edge in the graph. We extend this analysis to arbitrary discrete population models with first-order kinetics. The resulting decomposition allows us to rank the “importance” of each edge’s contribution to the variance of the current under stationary conditions. In most cases, transitions between open (conducting) and closed (non-conducting) states make the greatest contributions to the variance, but there are exceptions. In a 5-state model of the nicotinic acetylcholine receptor, at low agonist concentration, a pair of “hidden” transitions (between two closed states) makes a greater contribution to the variance than any of the open-closed transitions. We exhaustively investigate this “edge importance reversal” phenomenon in simplified 3-state models, and obtain an exact formula for the contribution of each edge to the variance of the open state. Two conditions contribute to reversals: the opening rate should be faster than all other rates in the system, and the closed state leading to the opening rate should be sparsely occupied. When edge importance reversal occurs, current fluctuations are dominated by a slow noise component arising from the hidden transitions.

## Author summary

Discrete state, continuous time Markov processes occur throughout cell biology, neuroscience, and ecology, representing the random dynamics of processes transitioning among multiple locations or states. Complexity reduction for such models aims to capture the essential dynamics and stochastic properties *via* a simpler representation, with minimal loss of accuracy. Classical approaches, such as aggregation of nodes and elimination of fast variables, lead to reduced models that are no longer Markovian. *Stochastic shielding* provides an alternative approach by simplifying the description of the noise driving the process, while preserving the Markov property, by removing from the model those fluctuations that are not directly observable. We previously applied the stochastic shielding approximation to several Markov processes arising in neuroscience and processes on random graphs. Here we explore the range of validity of stochastic shielding for processes with nonuniform stationary probabilities and multiple timescales, including ion channels with “bursty” dynamics. We show that stochastic shielding is robust to the introduction of timescale separation, for a class of simple networks, but it can break down for more complex systems with three distinct timescales. We also show that our related *edge importance measure* remains valid for arbitrary networks regardless of multiple timescales.

## Introduction

Variability in dynamical biological systems is ubiquitous. Discrete state, continuous time Markov process models are used throughout cell biology, neuroscience, and ecology to represent the random dynamics of processes transitioning among multiple locations or states [1–3]. Examples include transitions between states defined by degree of phosphorylation and subcellular compartment location in a signaling network [4], transitions among several conducting and non-conducting states in populations of ion channels [5], random genetic drift across a fitness landscape [6], random dispersal of mobile populations [7], and many other processes [8]. Often fluctuations arise at the molecular level, whether from discrete population effects, thermal (Brownian) effects, or deterministic high dimensional nonlinear dynamics (chaos) at microscopic scales.

In general, nonlinear stochastic systems cannot be solved mathematically in closed form. Even if we limit ourselves to Markov processes, *i.e.* models for which the probability distribution of future states is independent of the past history, given the current state (meaning that the current state is as complete a description of the process as possible, and no additional “hidden” variables exist), the effects of noise on biological dynamics must usually be studied via computer simulation. However, exhaustively simulating all noise sources within a given molecular level Markov process is often computationally prohibitive. Hence there is a need for complexity reduction methods.

**Fig 1:**
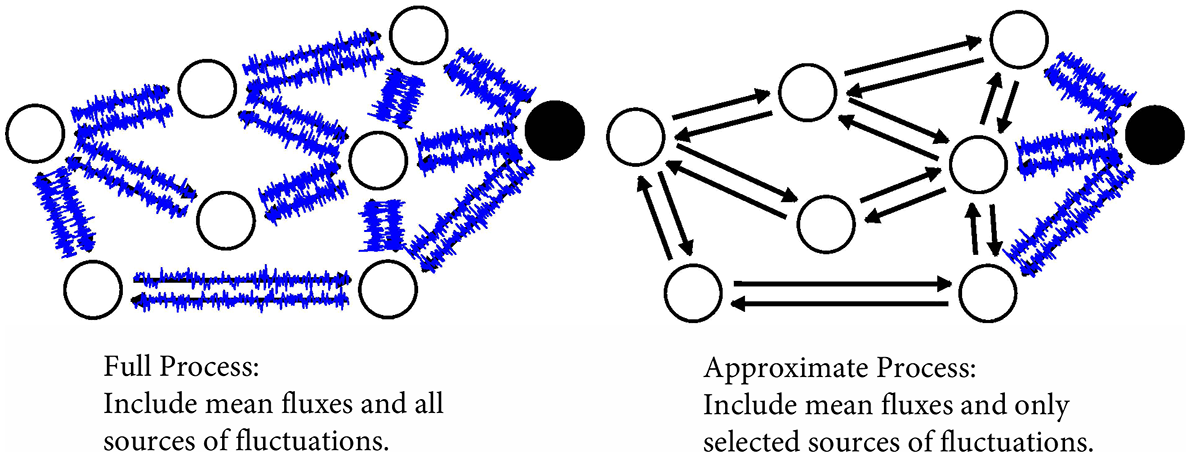
Schematic illustration of the stochastic shielding approximation, for a graph representing 24 transitions (directed edges) interconnecting eight states (vertices). One of the states (black disk) is distinguishable from the rest (white disks). For example, the black disk could represent a conducting ion channel state, while the white disks could represent non-conducting states. **Left:** Numerical simulation of the full process is computationally expensive: each blue trace superimposed on an edge represents independently generated stochastic forcing, but not all edges make significant contributions to fluctuations in the state of interest. **Right:** Rather than simulate the full process, the stochastic shielding approximation reduces the number of independent noise sources (blue edges) used to drive the stochastic process on the graph, while preserving the dynamical behavior of a particular projection of the random process.

In this paper we investigate a complexity reduction method for discrete state, continuous time Markov process models known as *stochastic shielding* which we summarize in the next paragraph [9, 10]. Complexity reduction for such models aims to capture the essential dynamics and stochastic properties of a system via a simpler representation, with minimal loss of accuracy. There is substantial literature on the approximation of complex random walk models with simpler models by mapping states of the full model to the nodes of a smaller set of states [11–23]. This includes coarse-graining of complex networks [11–13], elimination of fast variables *via* quasi-steady state approximation [24], marginalization of a partially observed Markov process through the solution of a filtering problem [25], the *k*-core decomposition (first proposed in [14] and shown to be effective for visualization in [15]), and various clustering algorithms that have been developed recently [16–20] (reviewed by [21]). Aggregation of tightly interconnected nodes and adiabatic elimination of fast variables lead to reduced models that are no longer Markovian [20, 22]. As another approach, one may eliminate rarely visited nodes, again leading to a reduction in the number of states [23]. *Stochastic shielding* provides an alternative approach by simplifying the description of the noise driving the process, while preserving the Markov property, by removing from the model those fluctuations that are not directly observable [9]. As illustrated in Fig 1, rather than reduce the number of nodes in the graph, the stochastic shielding approximation reduces the number of independent noise sources used to drive the stochastic process on the graph, while preserving the dynamical behavior of a particular projection of the random process.

As discussed in more detail in Methods §Summary of Stochastic Shielding in the Langevin Case, in the Langevin approximation for a time homogeneous first-order transition network, the population fraction occupying states 1, …, *n* is a vector $X\left(t\right)\in R^{n}$ satisfying

$$\begin{array}{c} \frac{dX}{dt}=LX+\sum_{k\in E}B_{k}\xi_{k} \end{array}$$ (1)

where *L*, the graph Laplacian, captures the mean flux along each directed edge k∈E (edge set). The matrix *B*_*k*_ gives the effects of fluctuations *ξ*_*k*_ around the mean flux along the *k*^th^ edge. The noise terms, *ξ*_*k*_, are independent, white and Gaussian, one for each directed edge. Given an observable of interest, represented by a vector $M\in R^{n}$, the stochastic shielding approximation consists in finding a partition of the edge set into edges of *primary* importance ($E_{1}$) and *secondary* importance ($E_{2}$) that gives an approximate process $Y\left(t\right)\in R^{n}$ satisfying

$$\begin{array}{c} \frac{dY}{dt}=LY+\sum_{k\in E_{1}}B_{k}\xi_{k},\;Y\left(0\right)=X\left(0\right) \end{array}$$ (2)

by neglecting the noise forcing along the edges of secondary importance. Such an approximation typically creates a (small) pathwise discrepency relative to **X** that can be quantified by our *edge importance measure*, also defined in Methods and discussed in more detail below [10].

The stochastic shielding approximation exploits filtering properties intrinsic to any network. Given an observable defined on the network (for example the indicator function for a subset of states representing nodes of interest), the fluctuations in population flux along some edges will have a greater impact on fluctuations in the observable, while other edges’ fluctuations will have a lesser impact. Hence the network “shields” the observable from some fluctuations, which may therefore be ignored with little loss of accuracy. To put it another way, the effects of a fluctuation in the movements of populations far removed from a location of interest do not directly affect the fluctuations in the population of interest; their effect reaches the observed nodes only via the indirect effect of influencing the population immediately surrounding the node or nodes of interest. One may view the source of fluctuations (relative to the average flux along a given edge) as independent noise forcing associated with each edge in the graph [10]. Edges that connect nodes that are indistinguishable, with respect to the measurement vector **M**, are themselves not directly observable. The fluctuations in rates of transition along these hidden edges are “averaged over” and their effect on the observed value (**M**^⊺^ **X**(*t*)) is reduced.

This filtering effect leads to the possibility of a novel approximation scheme. Rather than approximating a random process on a graph by aggregating together subsets of nodes, we may replace the fluxes along a subset of edges with the mean flux along the respective edge. If a graph has *K* directed edges, there are 2^*K*^ − 1 such “approximations”, as the independent noise along each edge can be either included or excluded from the approximation. Including all noise terms gives the original model, whereas excluding all noise terms gives a model with no fluctuations.

Which of these 2^*K*^ − 1 different approximations is the “best approximation”? The stochastic shielding *method* provides the following rule: suppress the noise along those edges connecting indistinguishable nodes. We extended this method by introducing an *edge importance measure* that quantifies the effect of suppressing noise along each edge separately. For a linearized Langevin equation (multidimensional Ornstein-Uhlenbeck (OU) process) approximating the full population process, we showed that when the process satisfies detailed balance, the variance of the observable states can be decomposed into a sum of fluctuations attributable to each pair of directed edges in the graph. Thus, the edge importance measure allows one to rank the edges such that the most important edge contributes the most to the stationary variance of the observable states.

We previously applied the stochastic shielding method to Markov processes arising in neuroscience (Hodgkin and Huxley’s sodium and potassium ion channel models) and processes on Erdos-Renyi random graphs [10]. However, these processes do not include significant timescale separation. In the present paper we study processes with nonuniform stationary probabilities and multiple timescales, including ion channel models with “bursty” dynamics.

Separation of timescales is an important property of many neural systems [26]. For instance, many ion channels exhibit bouts of repeated channel opening and closing, interspersed by long periods of channel closure—often referred to as bursty conductances. The nicotinic acetylcholine receptor (nAChR) is a well studied ligand-gated ion channel that can exhibit bursty behavior [27–29]. Acetylcholine (ACh) is a neurotransmitter that plays a key role in motor function *via* this ion channel, and the opening of the nAChR channel pore requires the binding of ACh. For low acetylcholine concentration ([ACh]), the nAChR is a classic example of a bursty ion channel.

In the next section, we explore the robustness of the stochastic shielding phenomenon and the accuracy of the approximation under conditions of timescale separation and sparsity in the stationary distribution, by way of the edge importance measure described in [10]. We show that typical edge importance hierarchy is robust to the introduction of timescale separation for a class of simple networks, but that it can break down for more complex systems with three or more distinct timescales, such as the nAChR described above. Nevertheless we also establish that the edge importance measure remains a valid tool for analysis for arbitrary networks regardless of multiple timescales.

## Results

### Overview

The shielding phenomenon leads the fluctuations associated with directly observable transitions to dominate the variance of the observable states in many networks, but this rule does not hold universally. The edge importance measure (see Eq 42 in Methods) provides an exact means to evaluate the applicability of stochastic shielding to any model (Markovian, with first-order transitions) by quantifying the effect of suppressing noise along each edge separately. This measure considers the pathwise mean square error between two trajectories: the full stochastic process with all fluctuations included, and an approximate process with a subset of fluctuations excluded. We use this measure to rank the edges in order of importance with respect to the stationary variance of the observable states. Moreover, we show that the stationary variance decomposes into a sum of contributions from each edge. This decomposition is unique and follows from a straightforward calculation that we describe and prove in Theorem 1 in the last subsection of Results. We apply the stochastic shielding method and compute the edge importance measure for the acetylcholine receptor model introduced above and for a set of simple networks (3-state chains) with timescale separation.

### Biological example of a bursty process: Nicotinic acetylcholine receptor

**Fig 2:**
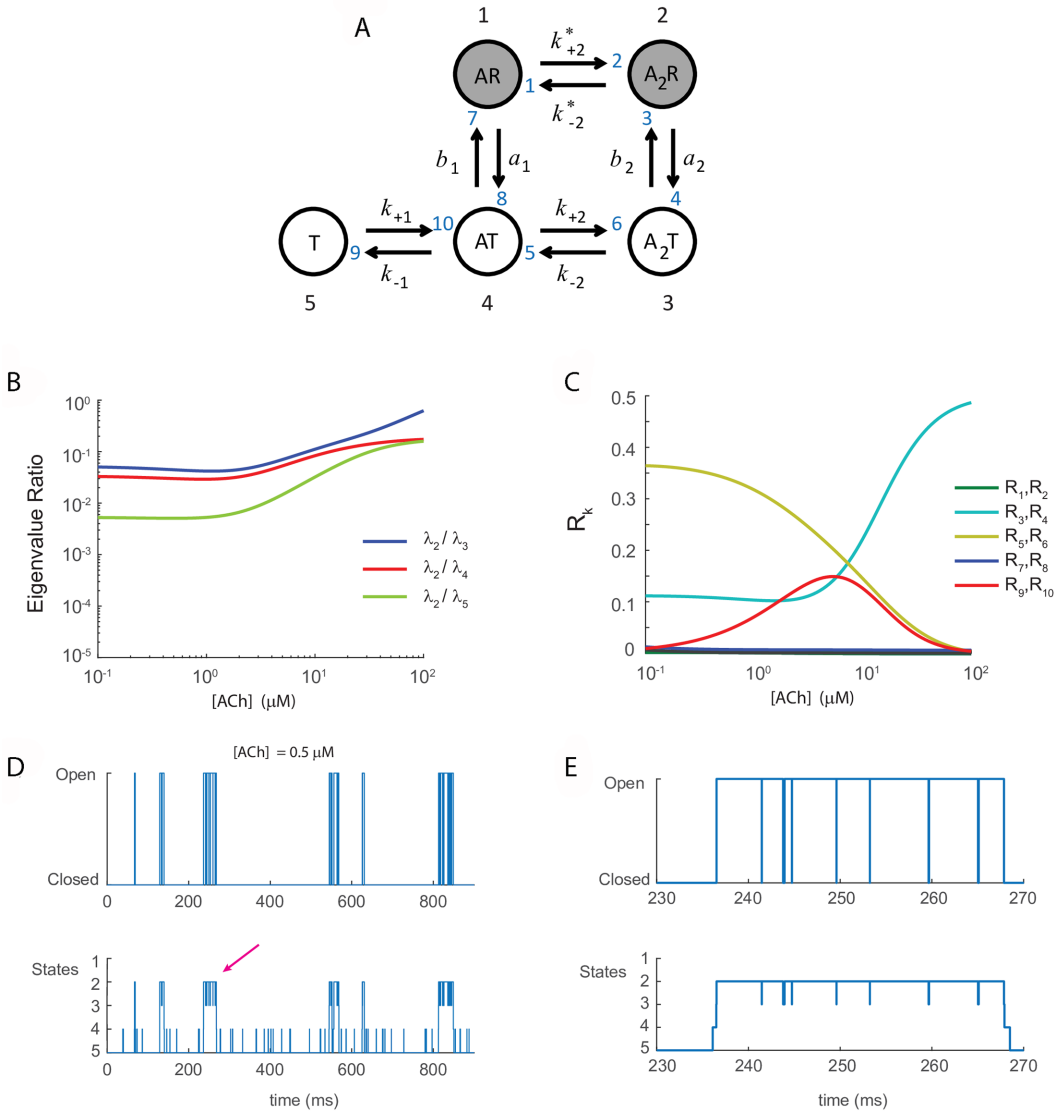
**(A)** Colquhoun & Hawkes’ five-state model for the nicotinic acetylcholine (ACh) receptor [30]. White disks: closed (non-conducting) states (*M*_*j*_ = 0). Gray disks: open (conducting) states (*M*_*j*_ = 1). Nodes 1-5 (large black numbers) are defined in Table 1. Transitions 1-10 (small blue numbers) are defined in Table 2. The opening of the channel requires the binding of acetylcholine. Transitions 2, 6, and 10 are driven by ACh concentration. Transitions 3, 4, 7, 8 are directly observable through a conductance change. **(B)** Timescale separation (ratio of non-zero eigenvalues of the graph Laplacian) as a function of ACh concentration. **(C)** Edge importance *R*_*k*_ for *k* ∈ {1, …, 10} for each edge in the graph as a function of [ACh], see Eq 42. **(D)** Sample trace of the model exhibiting burstiness of the channel for low agonist concentration, here [ACh] = 0.5*μ*M. **(E)** Zoomed in version of the burst in (D) labeled by the red arrow.

The nicotinic acetylcholine receptor is a ligand-gated ion channel and the opening of the channel pore requires the binding of acetylcholine. For low acetylcholine concentration, the nAChR is a classic example of a bursty ion channel. This channel has been described many times in the literature, and we will follow the formulation from Colquhoun and Hawkes [30]. Following Figure 4.1 in their paper, the channel has five states with ten possible transitions between states. The states form a graph with vertices i∈V={1,2,3,4,5} and edges k∈E={1,⋯,10} (see Tables 1 and 2). Fig 2A shows the transition state diagram. The channel can be bound to zero, one, or two ACh molecules. When singly or doubly bound the channel may be open or closed, whereas the unbound state is always closed. Table 1 gives the definition of the states and labels each state as open (observable) or closed (unobservable). State 5 (*T*) is the unbound state (closed), state 4 (*AT*) is singly bound (with 1 molecule of ACh) and closed, state 3 (*A*_2_*T*) is doubly bound and closed, state 2 (*A*_2_*R*) is doubly bound and open, and state 1 (*AR*) is singly bound and open. The measurement vector **M** specifies which states are open and which are closed by labeling each state with a 1 or 0, respectively. In this case, **M** is given by

$$\begin{array}{c} M={\left(\begin{array}{ccccc} 1 & 1 & 0 & 0 & 0 \end{array}\right)}^{⊺} \end{array}$$ (3)

meaning that states 1 and 2 are open/conducting states and states 3, 4, and 5 are closed/non-conducting states. Table 2 gives the definition of the edges and the transition rates. Note that the ten transitions are numbered starting with the pair of transitions connecting states 1 (*AR*) and 2 (*A*_2_*R*) and moving clockwise back to state 1; these are reactions 1-8. The last pair of transitions (9 and 10) connect states 4 (*AT*) and 5 (*T*). We will write the *per capita* transition rate for the *k*^th^ reaction, with source node *i* and destination node *j*, either with a single index denoting the reaction (*α*_*k*_) or with a double index denoting the source followed by the destination (*α*_*ij*_). Thus, *α*_1_ and *α*_21_ are synonymous.

**Table 1:** Colquhoun & Hawkes’ five-state model for the nicotinic acetylcholine receptor [30], *cf*. Fig 2A. Definition of the states and the measurement vector *M* (normalized conductance). *M*_*i*_ = 1 means that state *i* is open (conducting/observable) and *M*_*i*_ = 0 means that state *i* is closed (non-conducting/non-observable).

i∈V	State	*M*_*i*_
1	*AR* (singly bound and open)	1
2	*A*_2_*R* (doubly bound and open)	1
3	*A*_2_*T* (doubly bound and closed)	0
4	*AT* (singly bound and closed)	0
5	*T* (unbound and closed)	0

**Table 2:** Colquhoun & Hawkes’ five-state model for the nicotinic acetylcholine receptor [30], *cf*. Fig 2A. Definition of the edges and the transition rates *α*_*ij*_. The acetylcholine concentration is *c* ≥ 0. **Bold font** denotes edges with [ACh]-dependent transition rates.

k∈E	Transition	*i*(*k*) → *j*(*k*)	*α*_*ij*_
1	*A*_2_ *R* → *AR* (release)	2 → 1	$\alpha_{21}=2k_{-2}^{*}=0.\bar{6}\times 10^{-3}$
**2**	**AR → A**_**2**_ **R(binding)**	**1 → 2**	$\alpha_{12}=k_{+2}^{*}c=5\times 10^{-1}c$
3	*A*_2_*T* → *A*_2_*R* (opening)	3 → 2	*α*_32_ = *b*_2_ = 15
4	*A*_2_*R* → *A*_2_*T* (closing)	2 → 3	*α*_23_ = *a*_2_ = 0.5
5	*A*_2_*T* → *AT* (release)	3 → 4	*α*_34_ = 2*k*_−2_ = 4
**6**	**AT → A**_**2**_ **T(binding)**	**4 → 3**	**α**_**43**_ **= k**_**+2**_ **c = 5 × 10**^**−1**^ **c**
7	*AT* → *AR* (opening)	4 → 1	*α*_41_ = *b*_1_ = 1.5 × 10^−2^
8	*AR* → *AT* (closing)	1 → 4	*α*_14_ = *a*_1_ = 3
9	*AT* → *T* (release)	4 → 5	*α*_45_ = *k*_−1_ = 2
**10**	**T → AT(binding)**	**5 → 4**	**α**_**54**_ **= 2k**_**+1**_ **c** **= 10**^**−1**^ **c**

Burstiness is defined by the observation of isolated single channels opening and closing in bouts [29–31]. Fig 2D shows a sample trace of our model simulation exhibiting burstiness of the channel for low agonist concentration ([ACh] = 0.5*μ* Mol). (For details on the model simulation, see Numerical Methods, in Methods.) Fig 2E zooms in on the burst in panel D labeled by the red arrow. The distribution of closed intervals shows a mixture of slow and fast timescales, requiring combinations of two or more exponentials with widely separated time constants. These time constants are related to the eigenvalues of the graph Laplacians (see Eq 4, and see Methods for details). The ratio of eigenvalues will be used as a measure of timescale separation. Fig 2B shows the presence of timescale separation at low [ACh] concentrations by plotting the ratios of the eigenvalues {λ_2_/λ_*j*_}_*j* = 3,4,5_. Significant timescale separation occurs when λ_2_/λ_*j*_ << 1, or in words, when the two eigenvalues differ by at least one order of magnitude. The graph Laplacian has leading eigenvalue λ_1_ = 0. For the acetylcholine receptor, and for the systems we study here, the remaining eigenvalues are real and negative, and are ordered so that $0>\lambda_{2}\geq \lambda_{3}\geq \ldots \geq \lambda_{\left|V\right|}$, where |V| is the number of states.

We apply the stochastic shielding method to the nAChR model and show that it works well for high acetylcholine concentration, but not in the bursty regime characterized by low ACh concentration. In fact, we see a reversal of edge importance at low agonist levels (see Fig 2C and discussion below). In light of the network filtering effect underlying stochastic shielding, we might naïvely expect that the edges connecting states 2 and 3, and states 1 and 4, should contribute the most to the stationary variance of the observable states (1 and 2), but this is not the case. There is even a regime where the observable edge pair (edges 3 and 4) is only the third most important edge, as defined by our edge importance measure.

Computing the edge importance measure (Eq 42 in Methods), the fraction of the stationary variance contributed by edge *k*, requires the graph Laplacian *L* (and its corresponding eigenvalues and eigenvectors), the noise coefficient matrix *B* (defined below), the stationary mean flux *J*_*k*_, and the measurement vector **M**. The graph Laplacian *L* as a function of ACh concentration *c* is

$$\begin{array}{c} L=(\begin{array}{ccccc} -(a_{1}+k_{+2}^{*}c) & 2k_{-2}^{*} & 0 & b_{1} & 0\\ k_{+2}^{*}c & -(a_{2}+2k_{-2}^{*}) & b_{2} & 0 & 0\\ 0 & a_{2} & -(b_{2}+2k_{-2}) & k_{+2}c & 0\\ a_{1} & 0 & 2k_{-2} & -(b_{1}+k_{+2}c+k_{-1}) & 2k_{+1}c\\ 0 & 0 & 0 & k_{-1} & -2k_{+1}c \end{array}) \end{array}$$ (4)

and matrix *B* is

$$\begin{array}{c} B=(\begin{array}{cccc} \sqrt{J_{1}}\;\zeta_{1} & \sqrt{J_{2}}\;\zeta_{2} & \ldots & \sqrt{J_{10}}\;\zeta_{10} \end{array}), \end{array}$$ (5)

where *J*_*k*_ = *N*_tot_ *α*_*ij*_ *π*_*i*(*k*)_ is the stationary flux across edge *k* for a total population of *N*_tot_ ion channels, *α*_*ij*_ is the appropriate transition rate of reaction *k* (Table 2) and *ζ*_*k*_ is the stoichoimetry vector for reaction *k*. The *k*^*th*^ stoichoimetry vector describes how an individual moves from node *i* to node *j* in reaction *k*. For instance, the first two stoichoimetry vectors are

$$\begin{array}{ccc} \zeta_{1} & = & {\left(\begin{array}{ccccc} 1 & -1 & 0 & 0 & 0 \end{array}\right)}^{⊺} \end{array}$$ (6)

$$\begin{array}{ccc} \zeta_{2} & = & {\left(\begin{array}{ccccc} -1 & 1 & 0 & 0 & 0 \end{array}\right)}^{⊺} \end{array},$$ (7)

which correspond to transition 1 (an individual moves from state 2 to state 1) and transition 2 (an individual moves from state 1 to state 2), respectively, in Fig 2A. Note that *ζ*_1_ = −*ζ*_2_, and this relationship holds for each edge pair in the ACh transition graph.

The matrix *B* depends on the equilibrium population distribution $\vec{\pi }={\left(\pi_{1},\ldots ,\pi_{5}\right)}^{⊺}$. Since $\vec{\pi }$ is the leading eigenvector of the graph Laplacian *L*, the equilibrium fraction *π*_*i*_ of the population in state *i* will change as a function of *c* (ACh concentration). Lastly, recall that the measurement vector **M** = (1 1 0 0 0)^⊺^ as described in Table 1.

Fig 2C plots the relative edge importance *R*_*k*_ (fraction of the stationary variance contributed by edge *k*) for each edge *k* ∈ {1, …, 10} as a function of acetylcholine concentration over the range [ACh] ∈ [10^−1^, 10^2^] *μ*Mol. At high concentrations, the most important edges are those connecting the doubly bound closed state to the doubly bound open state (edges 3 and 4), that is, the edges along which transitions are directly observable. This situation is consistent with results for Hodgkin-Huxley ion channels and generic Erdos-Renyi random graphs with randomly assigned binary measurement vector [9, 10]. In contrast, the most important edges at low concentrations are those connecting the singly bound state to the doubly bound closed state (edges 5 and 6 in Fig 2A). Although transitions along this edge are *not* directly observable, they make a greater contribution to the stationary variance of the open state than the opening/closing transitions.

Moreover, we find that edges 5 and 6 have the highest relative importance for low and intermediate concentrations, followed by edges 3,4 and 9,10. Just below a concentration of 10 *μ*Mol, the relative importance switches so that edges 3 and 4 become the most important for higher concentrations (≥ 10 *μ*Mol). To begin to understand why the edge importance ranking changes for low [ACh], we note that the relative importance depends heavily on state occupancy probability.

As has been previously observed, one of the nodes in the 5-state nAChR model has very low occupancy probability across all agonist concentrations [32]. In particular, states 2 (*A*_2_*R*) and 5 (*T*) are the most likely states to be occupied over the range of [ACh] considered. However, state 1 (*AR*, one of the open states) has very low occupancy probability and hence is rarely visited by the process. As a result, the most likely path between the unbound/closed state 5 (*T*) and the doubly bound/open state 2 (*A*_2_*R*) is 5 → 4 → 3 → 2. This means that transitions 7,8 and 1,2 do not happen very often. The stochastic shielding method predicts that these reactions should be important, but if they rarely happen, they contribute little to the stationary variance. Thus, their relative importance as computed by our edge importance measure is very small. Indeed, for all values of [ACh], the equilibrium occupancy probability of state 1, *π*_1_ is ≪ 1. The variance of the open states for a population of *N*_tot_ channels at equilibrium is

$$\begin{array}{ccc} V[\text{Open}] & = & N_{\text{tot}}(\pi_{1}\left(1-\pi_{1}\right)+\pi_{2}\left(1-\pi_{2}\right)-2\pi_{1}\pi_{2})\\ & \approx & N_{\text{tot}}\pi_{1}\left(1-2\pi_{2}\right)+N_{\text{tot}}\pi_{2}\left(1-\pi_{2}\right)+O\left(\pi_{1}^{2}\right),\;\text{as}\;\pi_{1}\to 0^{+}. \end{array}$$

Although the goal of the stochastic shielding approximation is not to change the network topology by eliminating nodes as other authors have suggested [23, 32, 33], when edges are “unimportant” it is natural to consider eliminating them. If all the edges to a node are unimportant, eliminating them would eliminate the node, and in this case the change in stationary variance of the open states would be approximately *N*_tot_*π*_1_(1 − *π*_1_) − 2*N*_tot_*π*_1_*π*_2_, if *π*_1_ is small. (Compare to [32], “Scheme 1”.)

The edge importance measure *R*_*k*_ (for each edge *k*) provides an intrinsic idea of how many edges could be suppressed in an approximation (whether by suppressing the fluctuations generated by that edge, which is the focus here, or by removing the edge entirely). For the typical operating range of the nAChR, roughly 1-10 *μ*M [ACh], there are three transition pairs with similar edge importance (edge pairs 3,4, 5,6, and 9,10), suggesting that accurate simulations of stochastic effects would require keeping the fluctuations generated by all three of these edge pairs.

The acetylcholine receptor example suggests that the inversion of edge importance is related to timescale separation. In the next subsection, we investigate the edge importance measure in the presence of timescale separation, as well as a combination of sparsely and abundantly populated vertices. We show that edge importance ranking is preserved despite the introduction of arbitrary timescale separation in simple graphs (3-state chains) with *per capita* transition rates at two distinct timescales. As we will see, a system needs at least three distinct timescales in order to see the method break down. Nevertheless, the edge importance measure remains exact, and informative, for arbitrary networks, and can be used to extend the original stochastic shielding method to systems with timescale separation and bursty behavior.

### 3-state model with timescale separation

**Fig 3:**
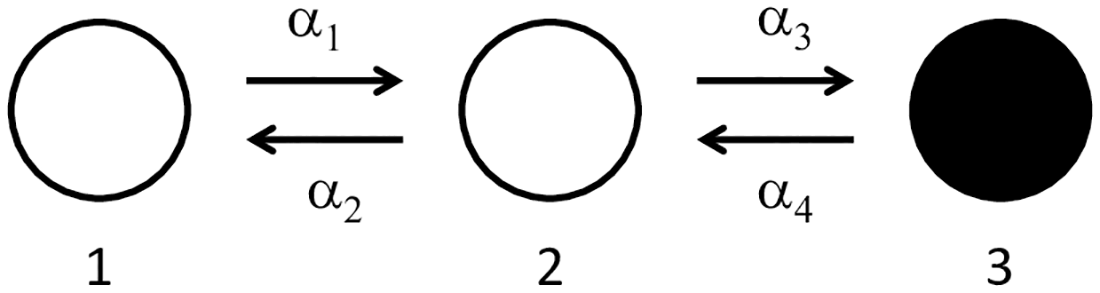
An illustration of the general 3-state chain with *per capita* transition rates *α*_*k*_ for *k* = 1, 2, 3, 4. State 3 (black) is the observable state (or open/conducting state) of the system, and all other states are not observable (or closed/non-conducting states). By convention, we identify *α*_1_ ≡ *α*_12_, *α*_2_ ≡ *α*_21_, *α*_3_ ≡ *α*_23_, and *α*_4_ ≡ *α*_32_.

Motivated by the example of the acetylcholine receptor, we systematically study the effects of introducing timescale separation into the simplest nontrivial model to which stochastic shielding applies: the 3-state chain with one observable state (or one pair of observable transitions into and out of the observable state). Specifically, we consider a discrete state, continuous time Markov jump process $N\left(t\right)\in N^{3}$ with *N*_tot_ random walkers moving independently on a graph with three nodes. See Fig 3 for an illustration of the graph, and see Methods for general notation and see S1 Supporting Information for a detailed description of the 3-state model. Here we assume that state 3 (black disk) is the observable state, which yields the following measurement vector: **M** = (0 0 1)^⊺^.

If we think of this model as a simplified ion channel with three states, then the observable state is the open or conducting state of the system, and all other states are closed or non-conducting. There are four directed edges in the graph, and edge *k* represents a transition from source node *i*(*k*) to destination node *j*(*k*) which happens at rate *α*_*k*_ (or *α*_*ij*_, see Methods for details on notation). We focus on the observed process **M**^⊺^**N**(*t*) which describes the evolution of the open state, and approximate processes that suppress noise along a subset of the four edges. In particular, we use the following two approximate processes to illustrate how stochastic shielding “usually” works: (i) suppress noise along edge pair 1,2 (and preserve noise along edge pair 3,4) and (ii) suppress noise along edge pair 3,4 (and preserve noise along edge pair 1,2). In most cases (i) is the best approximation; we investigate here whether or not this heuristic holds universally.

The mechanism of stochastic shielding can be readily understood by considering the power spectrum of the observed process **M**^⊺^**N**(*t*). The relationship between the power spectrum and the covariance matrix of a stochastic process is well known; the power spectrum is the Fourier transform of its covariance [34]. The stationary covariance *C* of a discrete state Markov process (such as **N** described above) is given by Gadgil, *et al.* [8], and satisfies the Lyapunov Eq 46 (see Methods).

The stationary variance *R* of the full and approximate observed processes has the following connection to the power spectrum: integrating over the power spectral density (PSD) *S*(*ω*) gives the stationary variance. Moreover, since the stationary variance decomposes into a sum of contributions from each edge in the graph (*R* = ∑_*k*_*R*_*k*_ where *R*_*k*_ is the edge importance measure of edge *k* given in Eq 42), the power spectrum decomposes as well (*S*(*ω*) = ∑_*k*_ *S*_*k*_(*ω*), see Eq 66). We provide more details on how the power spectrum is obtained in Methods §Numerical Methods.

**Fig 4:**
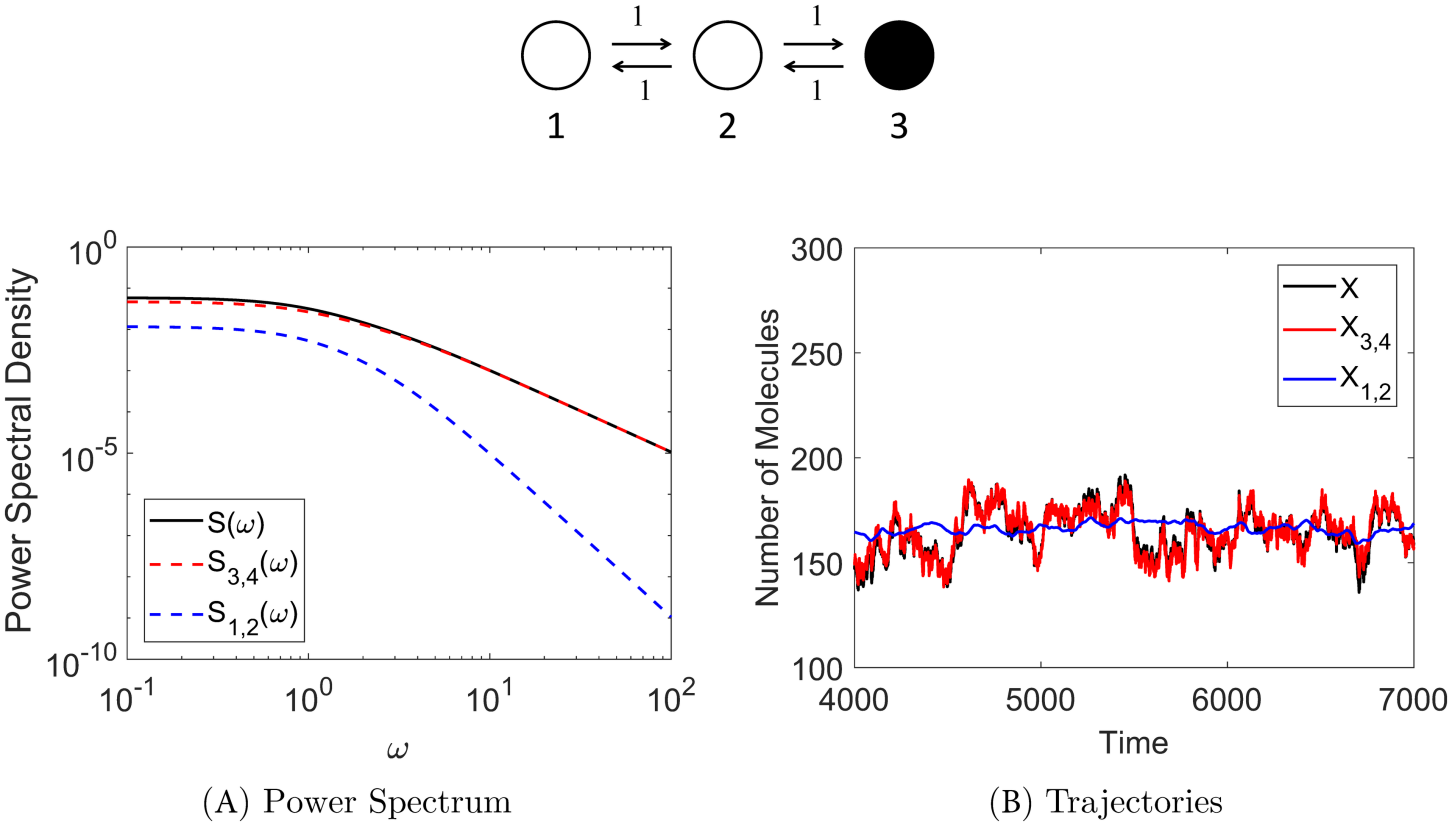
Stochastic shielding and the power spectrum when all transition rates equal unity. **Panel A** shows that the majority of the power comes from the observable edges (red dashed line), as expected from the edge importance measure and the stochastic shielding method. Black line is the total power spectral density (*S*) for the observed process *X*, red dashed line is the PSD (*S*_3,4_) for the approximate process *X*_3,4_ with noise from observable edges preserved, blue dashed line is PSD (*S*_1,2_) for the approximation *X*_1,2_ with noise from hidden edges preserved. **Panel B** shows trajectories (Gaussian version of the model) of the full observed process with all noise sources included (black trace), the approximate process with noise preserved on the observable edges (red), and the approximate process with noise preserved on the hidden edges (blue). The red trace closely follows the black trace, whereas the blue trace only tracks mean behavior and misses most fluctuations; *X*_3,4_ is the best approximation of *X*, in agreement with the stochastic shielding method.

Fig 4B shows sample trajectories for the full process (denoted by *X*, black trace) and the two approximations (i) and (ii) described above (denoted by *X*_3,4_ (red trace) and *X*_1,2_ (blue trace), respectively) in the Gaussian (OUP) version of the model. Fig 4A shows the corresponding power spectral contributions for the three processes: *S*(*ω*) is the total PSD (shown in black), *S*_3,4_(*ω*) is the PSD for approximation *X*_3,4_ (red), and *S*_1,2_(*ω*) is the PSD for approximation *X*_1,2_ (blue). See Methods §Numerical Methods for details on model simulation and calculation of the power spectra.

At all frequencies, the power from the observable edge pair 3,4 predominates, as shown by the red dashed line (*S*_3,4_(*ω*)) closely following the black line (total PSD). This spectral decomposition agrees with our edge ranking based on edge importance (i.e. edge pair 3,4 contributes the most to the stationary variance), and illustrates why the stochastic shielding method says that the best approximation of the full process is to preserve the noise along edge pair 3,4 and to suppress the noise along edge pair 1,2. Fig 4B illustrates the consequence in the time domain: the red trajectory closely follows the black trajectory, but the blue trajectory only captures a rough approximation of the full process.

However, this situation breaks down and leads to edge importance reversal for certain bursty systems, which we aim to understand in the rest of the paper. In the remainder of this section, we show that edge importance inversion cannot be obtained by taking a 3-state chain and accelerating or decelerating any single edge, pair, or trio of edges with a single parameter (*i.e.* by introducing two distinct timescales). As we shall see, in order to invert the edge importance as we did in the nAChR example for low agonist concentration, we need to introduce a third timescale. This will be addressed in §Generalized 3-State Model with Timescale Separation.

#### Two distinct timescales

Starting with two distinct timescales, we systematically survey all 3-state chains with one, two, or three out of four edge transition rates (*α*_*k*_) accelerated or decelerated relative to the remaining edges. The graph Laplacian *L* and matrix *B* for the 3-state model are given by

$$\begin{array}{c} L=(\begin{array}{ccc} -\alpha_{1} & \alpha_{2} & 0\\ \alpha_{1} & -\alpha_{2}-\alpha_{3} & \alpha_{4}\\ 0 & \alpha_{3} & -\alpha_{4} \end{array}),\;\;\;B=(\begin{array}{cccc} -\sqrt{J_{1}} & \sqrt{J_{2}} & 0 & 0\\ \sqrt{J_{1}} & -\sqrt{J_{2}} & -\sqrt{J_{3}} & \sqrt{J_{4}}\\ 0 & 0 & \sqrt{J_{3}} & -\sqrt{J_{4}} \end{array}) \end{array}$$ (8)

recalling that the stationary flux along the *k*^th^ edge is given by *J*_*k*_ = *N*_tot_*α*_*k*_*π*_*i*(*k*)_. The systems we consider satisfy detailed balance, which means that *J*_1_ ≡ *J*_2_ and *J*_3_ ≡ *J*_4_. Matrices *L* and *B* will be used to compute the relative edge importance *R*_*k*_ for each edge in all cases described below.

We introduce a parameter *α* (ranging from 10^−4^ to 10^4^) and consider seven different cases: three cases where two transition rates are set to *α* and the other two rates are 1, and four cases where one rate is set to *α* and the other three rates are 1. See Table 3 for a detailed description of these cases. In Case 1, transition rates between closed states differ from transition rates between the open and closed states; in Case 2, transition rates into the middle (closed) state differ from transition rates out of the middle state; in Case 3, upward transition rates differ from downward transition rates; in Cases 4-7, one of the four transition rates differs from the other three. We use Eq 42 (see Methods) to compute the relative edge importance *R*_*k*_ for each edge *k* as a function of *α*. The relative edge importance is computed with respect to the total variance of the third state.

**Table 3:** Detailed description of each case for the 3-state model. The first seven cases correspond to the chain that has the third state observable (see Fig 3). The last five cases have the middle state as the observable state. Transition rates *α*_*k*_ for *k* = 1, 2, 3, 4 are given by columns 3-6. The final column shows the characterization of each case into one of six different types determined by their edge importance graph as a function of parameter *α* (see right most column in Figs 5 and 6).

Case (1-12)	Observable State	Edge 1 → 2	Edge 2 → 1	Edge 2 → 3	Edge 3 → 2	Type (I-VI)
1	3	*α*	*α*	1	1	I
2	3	1	*α*	*α*	1	II
3	3	*α*	1	*α*	1	III
4	3	*α*	1	1	1	I
5	3	1	*α*	1	1	III
6	3	1	1	*α*	1	II
7	3	1	1	1	*α*	I
8	2	*α*	*α*	1	1	IV
9	2	1	*α*	*α*	1	V
10	2	*α*	1	*α*	1	VI
11	2	*α*	1	1	1	VI
12	2	1	*α*	1	1	IV

**Fig 5:**
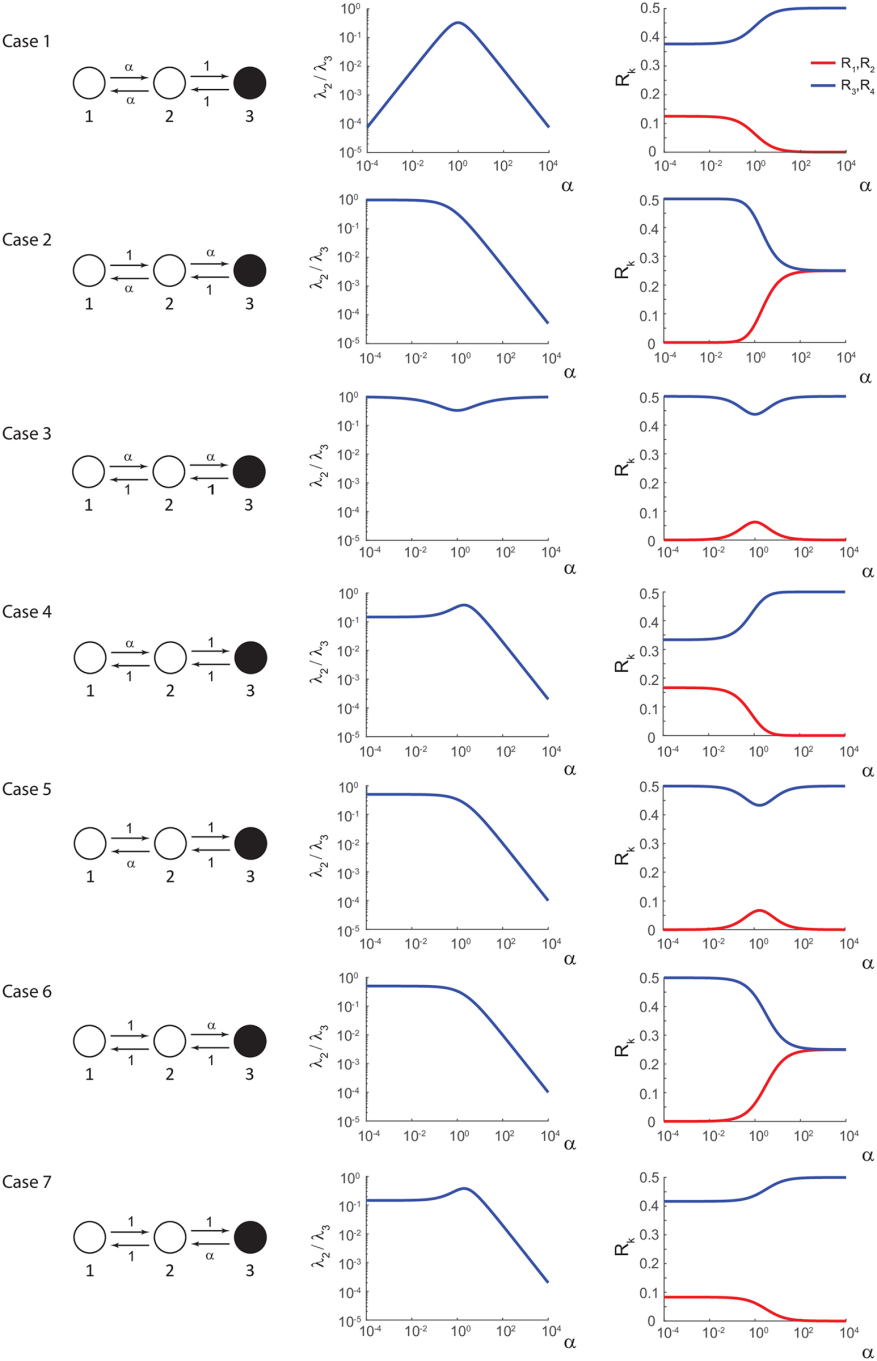
All possible cases for the 3-state chain with state 3 conducting. **Left column:** 3-state diagram with accelerated/decelerated edges labeled 1 or *α* where *α* ∈ [10^−4^, 10^4^]. **Middle column:** logarithm of the ratio of the two non-zero eigenvalues (λ_2_/λ_3_) versus *α*. This shows the “timescale separation” (present when λ_2_/λ_3_ ≪ 1). **Right column:** relative edge importance *R*_*k*_ versus *α*.

Fig 5 shows all possible cases of the 3-state chain with state 3 as the open/conducting state and *α* ranging from 10^−4^ to 10^4^. The left column gives the 3-state diagram for each case with transition rates *α*_*k*_ equal to 1 or *α*, as described in Table 3. The middle column shows the “timescale separation” for each case, defined to be the logarithm of the ratio of the two non-zero eigenvalues (λ_2_/λ_3_) versus *α*. As in the nAChR model, significant timescale separation occurs when λ_2_/λ_3_ ≪ 1, or when the two eigenvalues differ by at least one order of magnitude. The right column shows the relative edge importance *R*_*k*_ versus *α* for each case.

According to the stochastic shielding method, we expect edge pair 3,4 to have higher relative edge importance than edge pair 1,2. In Fig 5, this is shown by the blue curve (*R*_3_, *R*_4_) lying above the red curve (*R*_1_, *R*_2_) in the edge importance graphs in the right column. This holds for all cases except cases 2 and 6 which show a convergence of the two edge importance curves for large values of *α*. In these cases we also find significant timescale separation for large *α*, as shown by widely separated eigenvalues of the graph Laplacian (middle column graphs). For instance, when *α* = 10^4^, Cases 2 and 6 show that λ_2_ differs from λ_3_ by four orders of magnitude. However, there are several cases for which we find significant timescale separation but do not see a convergence or reversal of edge importance. Therefore, timescale separation is a necessary but not sufficient condition to see a breakdown of the stochastic shielding phenomenon.

Cases 2 and 6 share a feature that distinguish them from the other cases: although the importance of the hidden edges never exceeds that of the observable edges, they become equally important in the limit *α* ≫ 1. In both cases, this “fast” rate applies to the transition from the closed states to the open state (2 → 3). In Case 6, the 2 → 3 transition has the only accelerated rate; in Case 2 the 2 → 1 transition is also accelerated. However, in Case 3, which accelerates both the 2 → 3 and the 1 → 2 transitions, the edge importance does *not* converge. This conundrum will be resolved when we consider the general 3-state model (*cf.* Eq 22).

### 3-state model with middle state conducting

**Fig 6:**
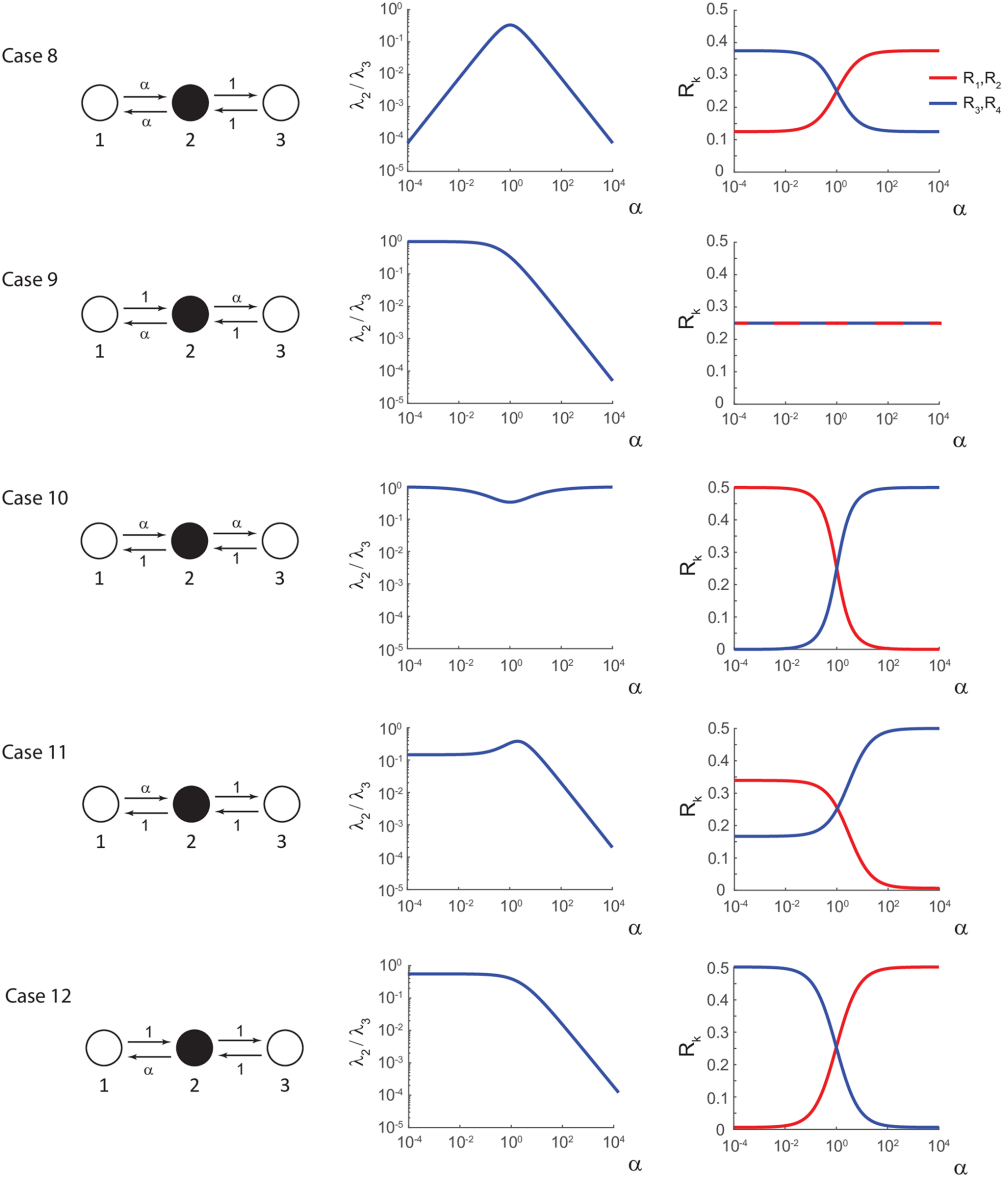
All possible cases for the 3-state chain with middle state 2 conducting. In this case there are no hidden transitions, and hence no stochastic shielding effect. **Left column:** 3-state diagram with accelerated/decelerated edges labeled as 1 or *α* where *α* ∈ [10^−4^, 10^4^]. **Middle column:** logarithm of ratio of the two non-zero eigenvalues (λ_2_/λ_3_) versus *α*. This shows the timescale separation (present when λ_2_/λ_3_ ≪ 1). **Right column:** relative edge importance *R*_*k*_ versus *α*.

For completeness, we may consider the same 3-state chain as in Fig 3, except that we set the middle state (state 2) to be the open/conducting state instead of state 3. The measurement vector in this case is **M** = (0 1 0)^⊺^. See the left column of Fig 6 for an illustration, and note that there are five possible cases to consider. In this version of the 3-state chain, all transitions are observable since each edge connects the conducting state to a closed state, and hence, all edges should be important in terms of stochastic shielding. State 2 no longer acts as a “shield” as it did when state 3 was the conducting state. We expect that the most important edges will either depend on the parameter *α* or all edges will be equally important in terms of the edge importance measure. We repeat the same analysis as in the previous section and the results are shown in Fig 6.

Fig 6 has the same three column format as Fig 5. The left column shows the 3-state diagram with accelerated/decelerated transition rates (1 or *α* as outlined in Table 3) where again *α* ∈ [10^−4^,10^4^]. The middle column shows timescale separation as defined by the ratio of the two non-zero eigenvalues (λ_2_/λ_3_) versus *α*. The right column shows the relative edge importance *R*_*k*_ versus *α*. In contrast to the previous cases with state 3 conducting, now we see edge importance reversal or convergence in every case. This is what we expect, given that the stochastic shielding method says that all edges are important in this version of the model. However, we find edge importance reversal in Case 10 without corresponding timescale separation since λ_2_ and λ_3_ differ by less than one order of magnitude in that case.

### Generalized 3-state model with timescale separation

We showed above that the presence of two distinct timescales was not sufficient to see an inversion of the edge importance in a 3-state network. However, as we show next, a network exhibiting *three* separate timescales can lead to edge importance reversal. In order to find examples of inversion, we study an ensemble of 3-state chains with observable state 3 (see Fig 2) with arbitrary transition rates {*α*_12_, *α*_21_, *α*_23_, *α*_32_}. We randomly draw the transition rates *α*_*ij*_ independently from a lognormal distribution with a given width *w*, that is, log(*α*_*ij*_) is Gaussian distributed with mean zero and standard deviation *w*. Then we calculate the edge importance for each realization of transition rates for this general 3-state model and look at the instances for which *R*_12_ = *R*_21_ > *R*_23_ = *R*_32_. Note that *R*_*ij*_ refers to the importance measure for the edges connecting node *i* to node *j*.

For an ensemble of 10^5^ samples with $\log\left(\alpha_{ij}\right)∼N\left(0,10\right)$ (i.e. $w=\sqrt{10}$), we find that inversion of the edge importance occurs approximately 9.8% of the time. This observation raises a number of questions. Which factors contribute to inversion of the usual edge importance relation (*e.g.* timescale separation)? Given an arbitrary set of transition rates, is there a canonical transformation leading to edge importance reversal? Can we obtain an exact expression for the relative contribution of the hidden edges to the stationary variance? The balance of this section addresses these questions.

**Fig 7:**
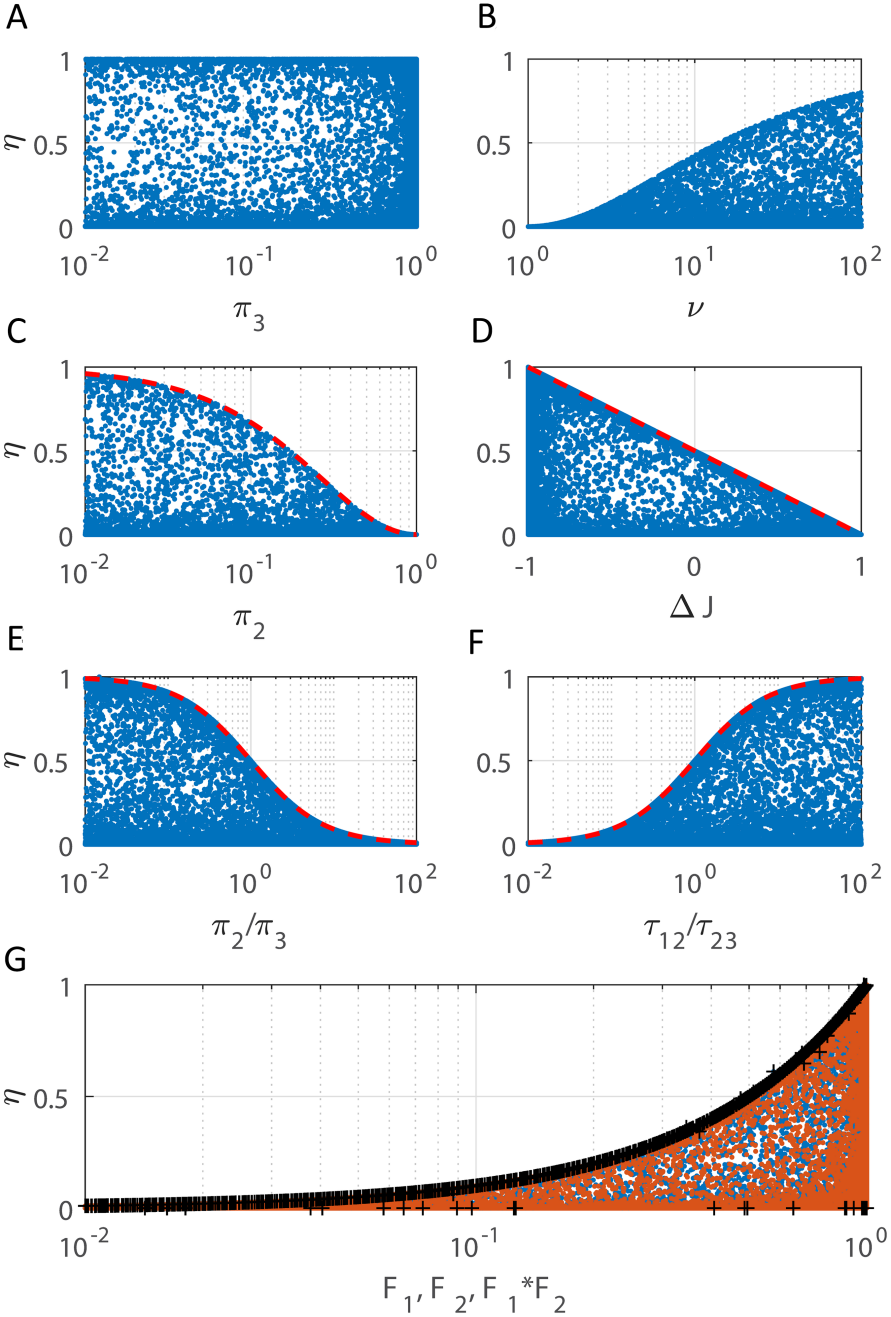
Factors contributing to edge importance reversal. The relative importance due to the hidden edges, *η* = *R*_12_/(*R*_12_ + *R*_23_), was calculated for an ensemble of 3-state chains (100,000 samples, see text for details). Relative edge importance is inverted when *η* > 0.5. **Left column** shows *η* plotted versus stationary occupancy probability of node 3 (*π*_3_, panel **A**), node 2 (*π*_2_, **C**), and the ratio of nodes 2 to 3 (*π*_2_/*π*_3_, **E**). The corresponding plot for *π*_1_ appears similar to that for *π*_3_ (not shown). Edge importance can be inverted for any values of *π*_1_ and *π*_3_, but requires *π*_2_ ≲ 1/6. **Right column** shows *η* plotted versus timescale separation (*ν* = λ_3_/λ_2_, **B**), relative fraction of flux generated by the hidden edges (Δ*J* = (*J*_12_ − *J*_23_)/(*J*_12_ + *J*_23_), **D**), and ratio of relaxation times for isolated 2-state systems corresponding to the hidden versus observable transitions (*τ*_12_/*τ*_23_, **F**). Edge importance reversal requires timescale separation (|λ_3_| ≳ 15|λ_2_| or *ν* ≳ 15), larger mean flux along the observable edges than the hidden edges (*J*_23_ > *J*_12_), and faster relaxation along the visible edges than along the hidden edges (*τ*_12_ > *τ*_23_). None of these conditions alone are sufficient. However, panel **G** shows *η* versus the two factors *F*_1_ and *F*_2_ in the exact expression for *η*, black ‘+’ line is *F*_1_ ⋅ *F*_2_ (see Eq 22).

Fig 7 illustrates the distribution, for this ensemble, of several factors that might be expected to play a role in inverting edge importance. Each panel plots the relative importance of the hidden edges

$$\begin{array}{c} \eta =\frac{R_{12}}{R_{12}+R_{23}} \end{array}$$ (9)

*versus* factors representing node occupancy, timescale separation, flux distribution, and local timescale difference. Inversion of edge importance occurs when *R*_12_ > *R*_23_, that is, when *η* > 1/2.

**Node Occupancy:** The left column of Fig 7 plots *η* versus the stationary occupancy probability of each state: *π*_3_ for state 3 (panel A), *π*_2_ for state 2 (panel C), and the ratio *π*_2_/*π*_3_ (panel E). Panel A suggests that edge importance can be inverted for any values of *π*_3_ (*mutatis mutandis* *π*_1_), but panel C suggests that inversion requires *π*_2_ ≲ 1/6. Moreover, panel E indicates that inversion requires *π*_2_ < *π*_3_ (equivalently, *α*_23_ > *α*_32_ since *π*_2_/*π*_3_ ≡ *α*_32_/*α*_23_). Together, these conditions suggest that sparse occupancy of the hidden state directly connected to the observable state (relative to the occupancy of the observable state) contributes to inversion of edge importance. However, this condition alone is not sufficient, as shown by Example A below (see Examples subsection), for which the relative importance due to the hidden edges is *η* = 0.4132 < 0.5.

We can extract several strict inequalities relating *η* to properties of the 3-state process. Maximizing *η* with *π*_2_ fixed, we find

$$\begin{array}{c} \eta \leq {\left(\frac{1-\pi_{2}}{1+\pi_{2}}\right)}^{2}. \end{array}$$ (10)

Fig 7C shows this inequality is tight (dashed red curve superimposed on the dots matches the upper boundary). In panel E, maximizing *η* with *π*_2_/*π*_3_ fixed, we observe that

$$\begin{array}{c} \eta \leq 1-(\frac{\pi_{2}/\pi_{3}}{1+\pi_{2}/\pi_{3}})=\frac{\pi_{3}}{\pi_{3}+\pi_{2}}=\frac{\alpha_{23}}{\alpha_{23}+\alpha_{32}} \end{array}$$ (11)

(dashed red curve matching boundary), which shows that inversion (*η* > 0.5) is only possible if *π*_2_ < *π*_3_, or equivalently, *α*_23_ > *α*_32_. More extreme edge importance inversion requires a more extreme likelihood difference between the observable state and its neighbor or between the transition rates connecting these states.

**Timescale Separation:** We introduce two different notions of timescale separation. First, we define

$$\begin{array}{c} \nu =\lambda_{3}/\lambda_{2} \end{array}$$ (12)

which is the ratio of the two non-zero eigenvalues of the graph Laplacian *L*. This quantity is shown in Fig 7B where *η* is plotted versus *ν*. (Note *ν* is the reciprocal of the ratio used to define timescale separation in the previous 3-state model sections with two distinct timescales and discussed in Figs 5 and 6). Large timescale separation, defined *via* the eigenvalues of the graph Laplacian, occurs when *ν* ≫ 1. Specifically, Fig 7B shows that edge importance reversal requires timescale separation such that |λ_3_| ≳ 15|λ_2_| or (*ν* ≳ 15).

Second, we consider the relaxation time

$$\begin{array}{c} \tau_{ij}={\left(\alpha_{ij}+\alpha_{ji}\right)}^{-1} \end{array}$$ (13)

for an isolated 2-state Markov processes with rates *α*_*ij*_, *α*_*ji*_ between the nodes *i*, *j*. The ratio of two such local relaxation times gives an alternative measure of timescale separation within the network. Specifically, consider the two possible 2-state processes in our 3-state model (nodes 1-2 and nodes 2-3). In the first system (between nodes 1 and 2), the eigenvalues of the graph Laplacian are 0 and *α*_12_ + *α*_21_ = 1/*τ*_12_. Likewise, looking at states 2 and 3 as a 2-state Markov process yields eigenvalues 0 and *α*_23_ + *α*_32_ = 1/*τ*_23_. Fig 7F shows the dependence of *η* on the ratio of the non-zero eigenvalues for these two 2-state systems. Empirically, we see that

$$\begin{array}{c} \eta \leq \frac{\tau_{12}/\tau_{23}}{1+\tau_{12}/\tau_{23}}=\frac{\tau_{12}}{\tau_{12}+\tau_{23}} \end{array}$$ (14)

(dashed red curve in Fig 7F where *η* is plotted versus *τ*_12_/*τ*_23_). That is, inversion of the edge importance (*η* > 0.5) occurs only when equilibration along the hidden edges is slower than along the observable edges (*τ*_12_ > *τ*_23_).

**Stationary Flux Distribution:** Recall that the stationary flux along edge *k* is given by *J*_*k*_ = *N*_tot_*α*_*k*_*π*_*i*(*k*)_. We can also represent this term as *J*_*ij*_, the stationary flux from node *i*(*k*) to node *j*(*k*) (see §Notation in Methods). Here we define

$$\begin{array}{c} \Delta J=\frac{J_{12}-J_{23}}{J_{12}+J_{23}} \end{array}$$ (15)

which is the relative fraction of the stationary flux generated by the hidden edges. In Fig 7D, we observe that the upper boundary is given by

$$\begin{array}{c} \eta \leq \frac{1}{2}-\frac{\Delta J}{2} \end{array}$$ (16)

which says that edge importance reversal (*η* > 0.5) requires larger mean flux along the observable edges than along the hidden edges. In other words, the system needs to satisfy Δ*J* < 0 or *J*_12_ < *J*_23_.

#### Conditions for edge importance inversion

The results of our ensemble analysis suggest that in order to invert the edge importance, the following conditions must hold:

1 The channel opening rate must be significantly faster than the other transition rates. That is, *α*_23_ ≫ max{*α*_12_, *α*_21_, *α*_32_}. This condition introduces a “fast” and a “slow” timescale.
2 Of the “shielded” transitions, the rate away from the observable state must be significantly faster than the rate towards the observable state. That is, *α*_12_ ≪ *α*_21_. This condition introduces a third “super slow” timescale, and guarantees a low occupancy probability for the middle state.

Further examination of the stationary flux along the edges reveals a third condition that is implied by the the first condition:

3 The majority of the stationary flux must be along the visible edges rather than the hidden edges. That is, *J*_12_ < *J*_23_.

To see this, note that (see Methods for derivation of stationary occupancy probability and flux)

$$\begin{array}{ccc} J_{12} & = & \pi_{1}\alpha_{12}=\frac{\alpha_{12}\alpha_{21}\alpha_{32}}{Z} \end{array}$$ (17)

$$\begin{array}{ccc} J_{23} & = & \pi_{2}\alpha_{23}=\pi_{3}\alpha_{32}=\frac{\alpha_{12}\alpha_{23}\alpha_{32}}{Z} \end{array}$$ (18)

where *Z* = *α*_12_*α*_23_ + *α*_12_*α*_32_ + *α*_21_*α*_32_. Then

$$\begin{array}{ccc} J_{23}-J_{12} & = & \frac{\alpha_{12}\alpha_{23}\alpha_{32}}{Z}-\frac{\alpha_{12}\alpha_{21}\alpha_{32}}{Z}=\frac{\alpha_{12}\alpha_{32}}{Z}\left(\alpha_{23}-\alpha_{21}\right) \end{array}$$ (19)

$$\begin{array}{ccc} J_{23}+J_{12} & = & \frac{\alpha_{12}\alpha_{32}}{Z}\left(\alpha_{23}+\alpha_{21}\right) \end{array}$$ (20)

$$\begin{array}{ccc} \Rightarrow \frac{J_{23}-J_{12}}{J_{23}+J_{12}} & = & \frac{\alpha_{23}-\alpha_{21}}{\alpha_{23}+\alpha_{21}}. \end{array}$$ (21)

Condition 1 guarantees that *α*_23_ > *α*_12_, and hence, *J*_23_ > *J*_12_.

#### Examples

Our ensemble suggests that both conditions 1 and 2 are necessary to see a reversal of the edge importance. However, the following two examples show that one condition can hold without inverting edge importance.

- Example A: *α*_12_ = *α*_21_ = 1, *α*_23_ = 10, and *α*_32_ = 0.1. Condition 1 holds, but not condition 2 since the first two transition rates are equal. Note that *π*_2_ < *π*_3_ (specifically, *π*_2_/*π*_3_ = 0.01). The fraction of the stationary variance generated by the hidden edges is *η* = 0.4132 < 0.5 which means that the observable edges have higher edge importance than the hidden edges, in agreement with the stochastic shielding method (i.e. no edge importance reversal).
- Example B: *α*_12_ = 0.1, *α*_21_ = 1, and *α*_23_ = *α*_32_ = 10. Condition 2 holds, but not condition 1 since the last two transition rates are equal. The fraction of edge importance due to the hidden edges is *η* = 0.4308 < 0.5 which, again, means that there is no reversal of edge importance.

Moreover, it is straightforward to see that the simple inequalities *α*_23_ > max(*α*_12_, *α*_21_, *α*_32_), and *α*_12_ < *α*_21_, are not sufficient. When *α*_*ij*_ ≡ 1 we have *R*_12_ = 1/8 and *R*_23_ = 7/8; the edge importance varies continuously with the *α*_*ij*_ so there will be some neighborhood of (1, 1, 1, 1) satisfying the simple inequalities for which inversion does not occur. For instance, setting *α*_23_ = *β*, *α*_12_ = 1/*β*, and *α*_21_ = *α*_32_ = 1, we find the hidden edges contributing the majority of the variance to the observable state 3 when *β* ≳ 3.848, while inversion does not occur for *β* ≲ 3.847, although all values *β* > 1 satisfy the simple inequalities.

### Exact expression for *η*

Reproducing edge importance reversal in 3-state chain models is advantageous because such simple Markov models can be analyzed more completely than models with greater numbers of states [23]. Fortunately, explicit expressions may be derived for the eigenvalues and eigenvectors of the 3-state chain model which allows direct calculation of *η*, the fraction of the stationary variance generated by the hidden edges (see S1 Supporting Information §Explicit calculation of *η* for detailed derivation):

$$\begin{array}{c} \eta \equiv \frac{R_{12}}{R_{12}+R_{23}}=(\frac{\alpha_{21}}{\alpha_{12}+\alpha_{21}})(\frac{\alpha_{23}}{\alpha_{12}+\alpha_{21}+\alpha_{23}+\alpha_{32}})=(\frac{\pi_{1}}{\pi_{1}+\pi_{2}})(\frac{\alpha_{23}}{\text{Tr}[-L]}) \end{array}$$ (22)

where Tr[*L*] ≡ ∑_*i*_ *L*_*ii*_ is the trace of *L*.

The fraction in Eq 22 is a product of two factors (denoted by *F*_1_ and *F*_2_ and shown in Fig 7G for the ensemble). The first factor *F*_1_ is the ratio of the speed of transition from hidden state 2 to hidden state 1 (*α*_21_) to the sum of the transition rates between states 1 and 2. Equivalently, this is the proportion of time spent in hidden state 1 relative to hidden state 2. *F*_1_ approaches 1 as *α*_12_ decreases, which only occurs if condition 2 holds (*α*_12_ ≪ *α*_21_). The second factor *F*_2_ is the ratio of the opening transition rate (*α*_23_) to the sum of the four rates. This factor is large if and only if the opening rate is much faster than the other rates, and this is exactly condition 1 (*α*_23_ ≫ max{*α*_12_, *α*_21_, *α*_32_}). Together these *two* conditions bring about a reversal of edge importance (*η* > 0.5) in this simple scenario.

While the exact formula for the relative edge importance (22) applies only for the 3-state chain model considered here, we anticipate that analogous results may hold for more general Markov processes. We consider this question further in §Discussion.

#### Canonical transformation leading to edge importance reversal

In §Generalized 3-State Model with Timescale Separation, we posed the question: *Given an arbitrary set of transition rates, is there a canonical transformation leading to edge importance reversal?* That is, can we show that inversion holds asymptotically in the following sense: given any *α*_*ij*_ > 0, if we accelerate the transition to the open state while simultaneously decelerating the exit from the hidden state, can we guarantee that we will eventually have inversion, and pushing the ratios further, approach 100% inversion? The answer is yes, and the exact expression (22) allows us to show this.

Given an arbitrary initial set of transition rates $\alpha_{ij}^{0}>0$ and *ϵ* > 0, define the following rescaled transition rates

$$\alpha_{12}=\epsilon \alpha_{12}^{0}\;\left(\text{decelerate}\;\text{transition}\;1\to 2\right)$$ (23)

$$\alpha_{21}=\alpha_{21}^{0}$$ (24)

$$\alpha_{23}=\frac{1}{\epsilon }\alpha_{23}^{0}\;\left(\text{accelerate}\;\text{transition}\;2\to 3\right)$$ (25)

$$\alpha_{32}=\alpha_{32}^{0}.$$ (26)

In S1 Supporting Information §Explicit calculation of *η*, we show that as *ϵ* approaches 0, we eventually reverse the edge importance and then approach *η* = *R*_12_/(*R*_12_ + *R*_23_) → 1. At the same time *π*_2_ → 0, the timescale separation grows, as does the flux imbalance. In other words, an arbitrarily large fraction of the stationary variance in the occupancy of the third state arises from the fluctuations in the transitions between nodes 1 and 2.

In summary, inversion of edge importance requires two conditions: the observable state dominates its immediate neighbor (higher occupancy probability), with which it rapidly comes to equilibrium, and this neighboring state is connected to a third state by a slow hidden transition. This combination results in a low occupancy of the intermediate state, but at the same time a larger stationary flux along the observable edges than along the hidden edges. These two conditions also explain why inversion occurs in roughly 10% of cases in our ensemble. To construct the ensemble we chose the edge weights *α*_*ij*_ to be independently and identically distributed (*iid*). Given four *iid* random variables *W*, *X*, *Y* and *Z*, it is an elementary exercise to show that *P*[{*Z* > max(*W*, *X*, *Y*)} ∩ {*W* < *X*}] = 1/8. Identifying *α*_12_ = *W*, *α*_21_ = *X*, *α*_32_ = *Y*, *α*_23_ = *Z*, and considering that the two conditions above are necessary but not sufficient, we expect the frequency of edge importance inversion to be less than, but not too different from, 12.5%.

Edge importance is a useful measure for evaluating the stochastic shielding effect that remains valid for arbitrary transition rates, despite the introduction of multiple timescales or sparsely occupied states. The stochastic shielding phenomenon occurs for a broad range of possible transition rates, with exceptions characterized by particular inequalities. Introducing a second timescale does not promote reversal of the edge importance, but introducing a second and third timescale to the graph dynamics in a specific way does.

### Edge importance and power spectra

**Fig 8:**
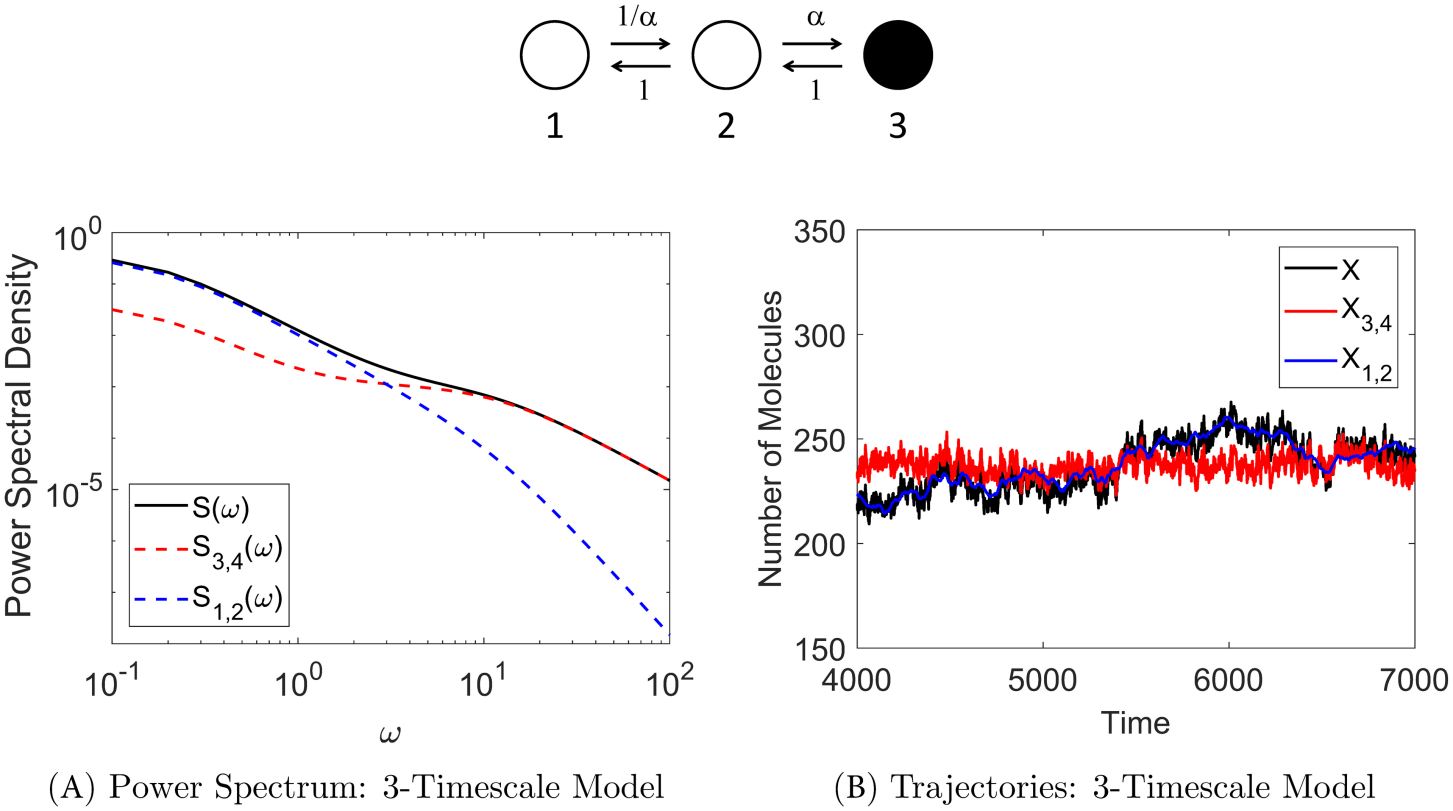
Stochastic shielding and the power spectrum in the 3-state chain for the case where edge importance is reversed (*α* = 10 shown here). **Panel A** shows that the majority of the power comes from the hidden edges (blue) for low frequencies, but the red and blue curves cross at *ω* ≈ 3, so for high frequencies the majority of the power comes from the observable edges (red). This switch in dominant spectral contributions is reflected in the corresponding Gaussian model trajectories shown in **Panel B** with the blue trace closely following the black trace, and the red trace deviating from the black trace. This shows that *X*_1,2_ best approximates the full process *X* in this case (whereas *X*_3,4_ is the best approximation in the uniform transition rate case shown in Fig 4).

Additional insight into the error arising from different noise-suppressing approximations can be obtained by examining the power spectral density distributions of the true and approximate processes. Recalling Fig 4A in the case *α*_*ij*_ ≡ 1, the power spectra for the full process with all noise sources included (*S*, black curve) and the approximate process with hidden edge flux noise suppressed (*S*_3,4_, red curve) are very similar, with an order of magnitude less power arising from the hidden edges at all frequencies. In contrast, Fig 8A shows the power spectra for the 3-timescale model. In particular, it shows that at low frequencies, the power spectrum for the approximate process with visible edge flux noise suppressed (*S*_1,2_, blue curve) is very similar to the PSD for the full process, but that the blue and red curves cross at an intermediate frequency (*ω* ≈ 3) so the red curve dominates at high frequencies.

The change in power spectral contributions is also reflected in model simulations (see Numerical Methods for details on simulations). Fig 8B illustrates sample trajectories for the three processes described above: full process *X* (black), approximation *X*_3,4_ (red), and approximation *X*_1,2_ (blue) where *α* = 10. Comparing this edge importance reversal case to the base case shown in Fig 4B, we see that the blue trajectory (instead of the red one) closely follows the black trajectory. Hence, *X*_1,2_ is the better approximation to the full process in this case.

Thus, the edge importance reversal observed under the combined conditions *α*_12_ ≪ *α*_21_ and *α*_23_ ≫ max(*α*_12_, *α*_21_, *α*_32_) may be understood as resulting from enhancement of the noise power contribution from the hidden edges at *low* frequencies, as well as the small amplitude of the full process’ power spectrum at *high* frequencies.

**Fig 9:**
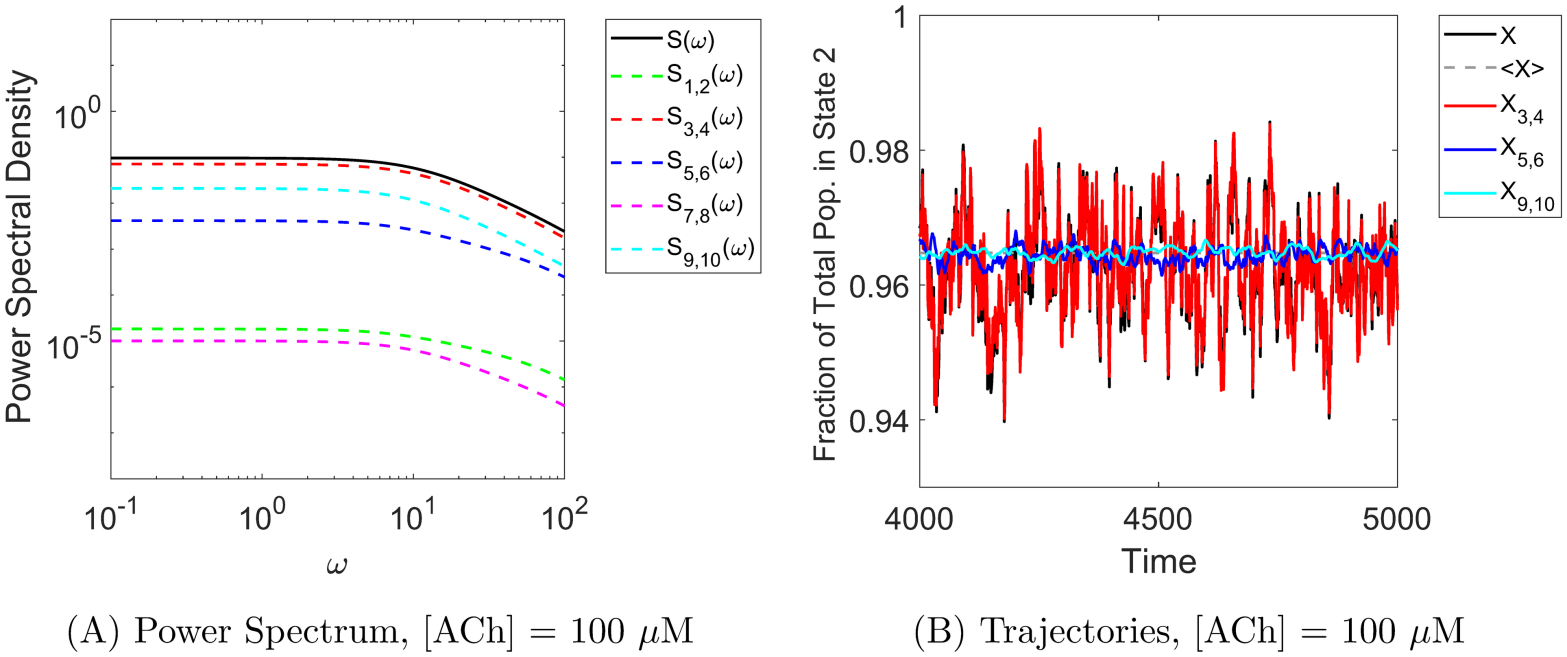
Stochastic shielding and the power spectrum in the acetylcholine receptor model. For a high concentration ([ACh] = 100 *μ*M shown here), edge importance is not reversed. **Panel A** shows that the majority of the power comes from the visible edges at all frequencies (*S*_3,4_, red trace), in agreement with the usual edge importance ranking. The corresponding Gaussian model trajectories shown in **Panel B** illustrate that *X*_3,4_ is the best approximation to *X*, whereas the other approximation only captures average behavior.

**Fig 10:**
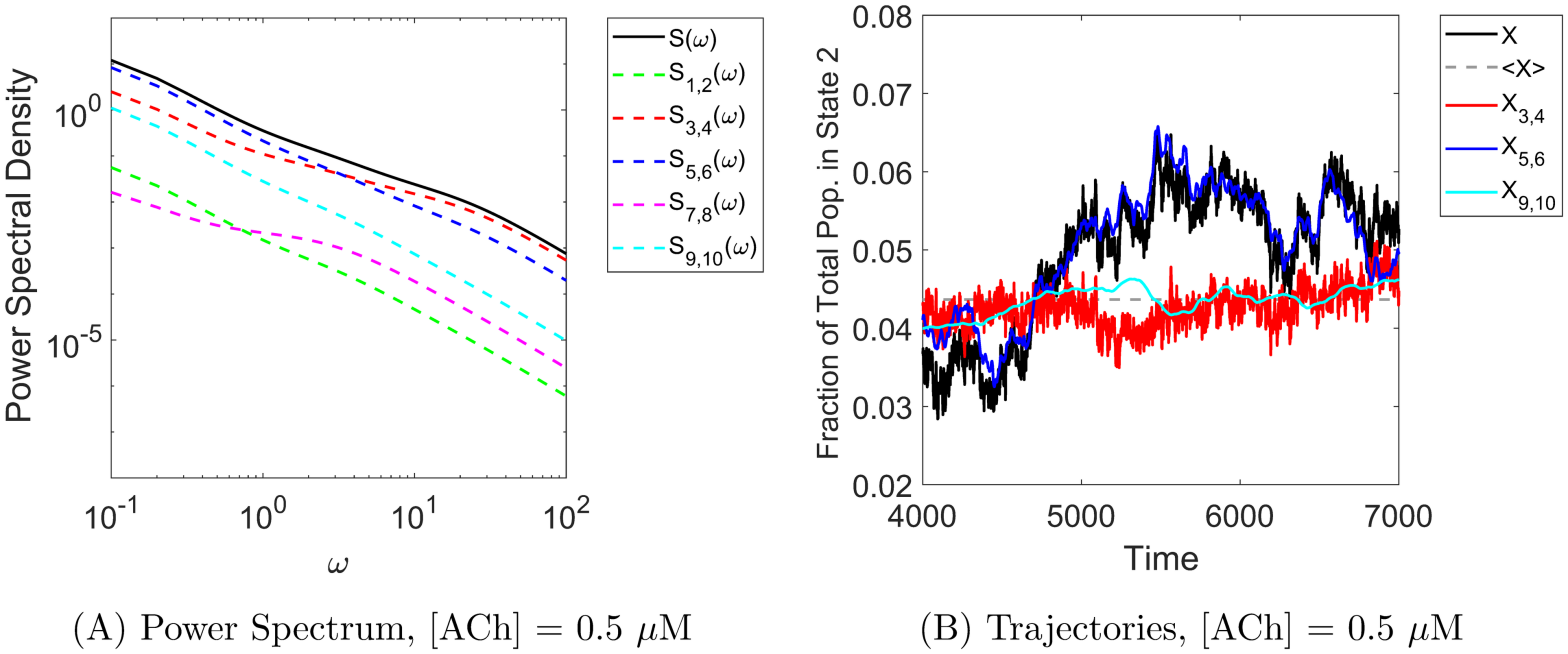
Stochastic shielding and the power spectrum in the acetylcholine receptor model. For a low concentration ([ACh] = 0.5 *μ*M shown here), edge importance is reversed. **Panel A** shows that the majority of the power comes from the hidden edges at low frequencies (*S*_5,6_, blue trace) and from the visible edges at high frequencies (*S*_3,4_, red trace), just as we saw in the 3-state model under the case of edge importance reversal. The corresponding Gaussian model trajectories shown in **Panel B** illustrate that *X*_5,6_ (blue) is the best approximation to *X*, although it misses some the fluctuations captured by *X*_3,4_ (red).

We see a similar mechanism at work in the 5-state acetylcholine receptor model in the low-[ACh] regime (where a hidden edge becomes more important than a visible edge) as opposed to the high-[ACh] regime, in which the usual edge importance ordering is observed. Figs 9 and 10 show the power spectrum and Gaussian model trajectories in the high-[ACh] and low-[ACh] regimes, respectively. Here we have similar notation to the 3-state cases: *X* (black) is the full observed process (Gaussian version) with all sources of noise included and *X*_*i*,*j*_ is the approximate process that preserves noise on edge pair *i*, *j* but suppresses noise on all other edges. In particular, we focus on the red trace (*X*_3,4_, noise preserved on visible edges 3,4) and the blue trace (*X*_5,6_ noise preserved on hidden edges 5,6).

The usual edge ordering via the edge importance measure for high [ACh] ranks edge pair 3,4 the most important, followed by edges 5,6, then 9,10 (the last two edge pairs have relative importance close to 0 and make the two lowest spectral contributions); See Fig 2. Fig 9A shows that for [ACh] = 100 *μ*M, most of the power is attributable to the observable edge pair 3,4, and this agrees with the edge importance ranking. Model trajectories in panel B illustrate that *X*_3,4_ is the best approximation of the full process *X* and that the other approximations at best only capture the mean behavior of the system.

In the low-[ACh] case shown in Fig 10 ([ACh] = 0.5 *μ*M), however, we see the crossing of the top blue and red power spectral density curves at an intermediate frequency (*ω* ≈ 2). As in the 3-state case, this indicates a reversal of edge importance whereby now the hidden edge pair 5,6 contributes the most to the stationary variance of the observable process. Again, this change in spectral contributions is reflected in model trajectories shown in panel B. We see that the blue curve *X*_5,6_ closely follows the full process *X*, and is the best approximation in this case, but the blue curve misses some of the fluctuations captured by the red curve *X*_3,4_ even though the red curve clearly deviates from the other two processes.

### Edge importance and decomposition of the stationary observed variance for a general first-order transition network

Gadgil *et al.* showed rigorously that the time evolution of the second moments of a discrete population evolving as a first-order reaction network system can be represented explicitly in terms of the eigenvalues and eigenvectors of the matrix that governs the evolution of the mean population dynamics [8]. We apply their general results to the specific example of a first-order transition network in two ways. First, we use the spectral decomposition of the stationary variance to establish our main stochastic shielding result. Second, their result on time varying systems allows us to obtain the decomposition of the power spectrum in terms of the eigenvalue spectral decomposition, shown in Eqs 64–66.

Consider an arbitrary first-order reaction network with graph Laplacian *L* and matrix *B* satisfying Eqs 32–36 (see Methods). The fact that the stationary covariance matrix decomposes into a sum of contributions from each edge in the graph follows from a straightforward calculation that we describe in Lemma 1 and Theorem 1. We defer the proof of Lemma 1 to §Methods, below.

**Definition 1** *Let X denote the set of n* × *n real matrices C such that for all j* = 1, …, *n*, $\sum_{i=1}^{n}C_{ij}=0$. *Let* *Y* = {*C* ∈ *X* | *C*^⊺^ = *C*}.

**Lemma 1** *Let L be an n* × *n real valued matrix with L*_*ij*_ ≥ 0 *for i* ≠ *j, and L*_*ii*_ = −∑_*i*,*i* ≠ *j*_ *L*_*ij*_ (*so that* $\sum_{i=1}^{n}L_{ij}=0$) *for j* = 1, …, *n, and satisfying dim*(*ker*(*L*)) = 1, *and with a null eigenvector Lv* = 0 *satisfying v*_*i*_ ≥ 0 *for i* = 1, …, *n. Then for any F* ∈ *Y, the equation*

$$\begin{array}{c} LC+CL^{⊺}=F \end{array}$$ (27)

*has a unique solution C* ∈ *Y*.

**Theorem 1** *For an arbitrary first-order reaction network with graph Laplacian L and matrix B satisfying* Eqs 32–36, *there is a unique linear decomposition of the stationary covariance matrix C as a sum of contributions from each edge*:

$$C=\sum_{k\in E}C_{k}\;where$$ (28)

$$C_{k}=\int_{0}^{\infty }\left(e^{tL}\right)B_{k}B_{k}^{⊺}{\left(e^{tL}\right)}^{⊺}dt$$ (29)

**Proof 1** *Proof of Theorem 1. Consider a first-order reaction network defined by graph Laplacian L and matrix B, satisfying* Eqs 32–36. *We want to solve the Lyapunov equation*

$$\begin{array}{c} LC+CL^{⊺}=-BB^{⊺} \end{array}$$ (30)

*for matrix C. Note that L satisfies the conditions in Lemma 1, and BB*^⊺^ ∈ *Y since BB*^⊺^ *is an n* × *n real symmetric matrix with columns that sum to zero. Then by Lemma 1*, Eq 30 *has a unique solution C* ∈ *Y. By replacing F with BB*^⊺^ *in the proof of Lemma 1, we see that the unique solution is*

$$C=\int_{0}^{\infty }e^{tL}BB^{⊺}{\left(e^{tL}\right)}^{⊺}dt$$ (31)

*since all eigenvalues of L have negative real part (except for the Perron-Frobenius eigenvalue* λ_1_ ≡ 0), $u_{1}^{⊺}B=0$, *and* *B*^⊺^*u*_1_ = 0.

*Since BB*^⊺^ *can be written as a sum of* $B_{k}B_{k}^{⊺}$ , *we can repeat the calculation above to get* Eq 29 *for each k separately. The integral in* Eq 29 *holds for all k since the k*^*th*^ *stoichiometry vector ζ*_*k*_ *appearing in the k*^*th*^ *column of B is orthogonal to the steady state eigenvector. Therefore, C decomposes into a sum over the C*_*k*_ *terms, and* Eq 28 *holds*.

The decomposition in Theorem 1 allows us to rank each edge in the network in terms of its contribution to the stationary variance of any given node, which we call its “importance” relative to that node. In the case of a single open or conducting node, we refer simply to the edge importance. Moreover, the decomposition allows us to quantify the accuracy of the stochastic shielding approximation with respect to the population process projected onto individual nodes. The decomposition given by Theorem 1 is exact regardless of timescale separation or node sparsity.

## Discussion

Markov chains provide a general framework for mathematically modeling and simulating stochastic processes in natural and artificial systems. However, Markov chains are computationally expensive as their simulations require random numbers at each time step for every transition (edge). The stochastic shielding approximation relies on the fact that, when hidden states are present, the edges are not equally important, so that random fluctuations in some (typically most) edges can be neglected. Here, we provide a thorough study addressing how to identify the relevant and irrelevant edges when the stochastic fluctuations span slow and fast timescales. Our analysis shows that the stochastic shielding approach not only provides a practical increase in computational efficiency, but also facilitates a systematic understanding of the propagation of fluctuations in a general Markovian network, and hence, is applicable to many areas of mathematical biology and related disciplines.

The stochastic shielding method is being used increasingly to incorporate fast, accurate simulation of stochastic ion channels into larger neuronal network models. A recent paper [35] comparing different methods for simulating ion channels, based on diffusion approximations, recommended using the stochastic shielding approximation in conjuction with a direct Langevin approach advanced by Orio and Soudry [36]. Two examples in which stochastic shielding makes large-scale simulations tractable include [37] and [38]. In the first paper, the use of stochastic shielding allowed for a significant reduction in computation time of multiple simulations of a conductance-based model with synaptic and ion channel noise that are necessary to reliably estimate the entropy and information rate of neuronal firing. In the second paper, stochastic shielding is applied to a heterogeneous neural circuit for the first time, allowing the authors to investigate the role of channel noise in the generation of breathing variability in the isolated central pattern generator of respiration. In both cases, these studies would have not been possible in practice without the stochastic shielding approximation.

The analysis conducted here and in [10] is restricted to the case of a stationary Markov process, *i.e.* with time-invariant *per capita* transition rates. In many applications, for example under current-clamp (rather than voltage-clamp) in electrophysiology, the transition rates vary over time. In [9], which introduced the stochastic shielding method, stochastic shielding was shown to produce accurate approximations through comparison of voltage traces and spike trains generated via both stochastic shielding and full Monte Carlo simulations.

In the present paper, we have shown that in the presence of multiple timescales, for instance as seen in the dynamics of the nicotinic acetylcholine receptor (nAChR) under low agonist concentrations, one or more unobserved edges can become more important than the observable edges, in terms of making a greater contribution to the stationary variance of the occupancy of the open channel state (and hence the variance of the ionic current through the population of channels). In such a case the stochastic shielding phenomenon is still present, but is significantly reduced, to the point that the approximation given by suppressing the noise on the hidden edges does not provide the best approximation. Indeed, as seen in Fig 10, one may conclude that in this situation there is no suitable approximation of the type we consider, since the traces generated by reduced models with noise suppressed either on the observed or unobserved edges do not bear much similarity to the trace generated by the full model (with identical noise forcing where the noise is included). On the one hand, the edge importance measure remains exact under all conditions, as long as the network is irreducible (meaning here that *α*_12_, *α*_21_, *α*_23_ and *α*_32_ are all nonzero). On the other hand, the stationary variance does not capture the full shape of the trajectories. The decomposition of the fluctuations at one node as a sum of contributions from distinct edges extends to the correlation function and the power spectrum and the cross-spectrum, as well as to the variance.

Motivated by the example of the nicotinic acetylcholine receptor, we systematically studied the effects of introducing separation of timescales into the simplest nontrivial model to which stochastic shielding applies: the 3-state chain with one observable state. We found that, in the case of two distinct timescales, accelerating or decelerating a subset of edges relative to a baseline case (*α*_*ij*_ = 1 for all adjacent nodes (*i*, *j*)) could in some cases enhance, and in other cases reduce the gap in edge importance between the observed and unobserved edges, but in no case could induce a reversal of the edge importance (as observed in nAChR).

Finally, by sampling an ensemble of different transition rates, we found that inversion of edge importance can be seen in a 3-state chain when the channel opening rate is large (that is, *α*_23_ ≫ max(*α*_12_, *α*_21_, *α*_32_)), *and also* the rate of return from the first hidden state to the middle hidden state is small (that is, *α*_12_ ≪ *α*_21_). These complementary conditions are captured by the exact expression for the relative edge importance (Eq 22). Together, these conditions lead to sparse occupation of the middle node, introducing a bottleneck, while also introducing timescale separation in such a way that equilibration between the observable node and its immediate neighbor occurs much faster than between the two unobservable nodes.

Although our exact formula applies only to the 3-state chain model from which it was derived, we are optimistic that it may be extended to broader classes of Markov processes. The forms of such extensions are not *a priori* obvious, for several reasons. Consider the case of an ion channel with *n* states of which a single open conducting state (*O*_*n*_) is connected to the closed, non-conducting states (*C*_1_, …, *C*_*n*−1_) through a single bottleneck state (*C*_*n*−1_); the closed states may interconnect arbitrarily with rates *α*_*ij*_, 1 ≤ *i*, *j* ≤ (*n* − 1). In this case the analog of the first factor in Eq 22 would be the conditional occupancy probability of the bottleneck node *C*_*n*−1_, given the channel is in any of the states *C*_1_, …, *C*_*n*−1_. However, the analog of the second factor, the ratio of the *C*_*n*−1_ → *O*_*n*_ transition to some combination of all the rates in the system, is far from clear. For ion channel models with multiple transitions into and out of a single open state (see Fig 1), the parallel to our exact 3-state chain analysis is scarcely obvious, and remains for future investigation.

The stochastic shielding approximation and method provide an approach distinct from aggregation based on community structure [20] or similarity of spectral components [13, 39], and pruning of sparsely populated nodes [23, 33], although there are some relations between these methods. Both spectral coarse graining [13] and our edge importance measure [10] rely on spectral decomposition of the graph Laplacian. As Ullah *et al.* point out, finding eigenvalues and eigenvectors of the Laplacian for a large complicated graph can be challenging [23]. An advantage of the stochastic shielding method is that it can be applied in the vast majority of cases without calculating the edge importance explicitly. Exceptions can occur when there is significant timescale separation with fast relaxation of the observable node with its immediate neighbors and slow relaxation among unobservable states, with a hidden bottleneck state separating the observable from a well populated pool of unobservable nodes. Except in this particular case, the stochastic shielding method can be applied without necessarily having to calculate the edge importance in detail. The effect of fluctuations in rates along the hidden edges is filtered by the network, and their impact on fluctuations at the observable nodes is diminished.

## Materials and methods

In this section we fill in the details behind the results. We introduce notation, define the edge importance measure relative to an arbitrary measurement vector, justify our use of the Lyapunov equation, prove Lemma 1, and describe our numerical methods.

In S1 Supporting Information, we establish the decomposition of the stationary variance. We provide explicit calculations for the 3-state process, and calculate *η*, the fraction of variance of the observable state arising from the hidden edges. We review the connection between the population process and Gaussian approximations thereof, and give a detailed derivation of the Lyapunov equation for the 3-state case.

### Notation

We begin with a directed graph G=(V,E) with edge weights *α*_*ij*_ ≥ 0 representing a population of *N*_tot_ individuals moving randomly and independently among *n* states (i,j∈V) along *m* edges {*i*(*k*)→*j*(*k*)}_1≤*k*≤*m*_, with *per capita* transition rates {*α*_*k*_}_1≤*k*≤*m*_. We emphasize that edge *k* is the unique directed edge connecting source node *i*(*k*) to destination node *j*(*k*). The *n* × 1 stoichiometry vector *ζ*_*k*_ corresponding to edge *k* is defined such that *ζ*_*k*_(*i*) = −1 and *ζ*_*k*_(*j*) = +1, otherwise *ζ*_*k*_(*l*) = 0; these vectors represent the effect of a transition along edge *k*. We use this notation to be consistent with the edge importance formula in the next subsection which is a sum of contributions to the variance of the observable state coming from each edge. Also, note that we will write the *per capita* transition rates either with double indexing denoting the source and destination nodes (*α*_*ij*_) or with a single index denoting the reaction (*α*_*k*_).

We represent the population state at time *t* with an integer-valued vector **N**(*t*) = (*N*_1_(*t*), …, *N*_*n*_(*t*))^⊺^, where *N*_*i*_(*t*) ≥ 0 and $\sum_{i=1}^{n}N_{i}\left(t\right)=N_{\text{tot}}$ for all *t*. In other words, **N**(*t*) is a discrete state continuous time Markov process. Such processes are ubiquitous in biology [1].

We denote by **M** a *measurement vector* indicating a direction in the state space along which there is an observable of interest. For instance, *M*_*i*_ ∈ {0, 1} could denote the conducting state ({closed, open}) in a multi-state ion channel model. We denote the observed process by *Y*(*t*) = **M**^⊺^**N**(*t*). The remainder of our set up follows standard nomenclature for representing a population process on a graph [8, 40–42].

Let *L* be the Laplacian of graph G which is the *n* × *n* matrix defined by *L* = (*A* − *D*)^⊺^ where *A* is the weighted adjacency matrix and *D* is the diagonal matrix of node out-degrees. Specifically, the entries in *A* are *A*_*ij*_ = *α*_*ij*(*k*)_ = *α*_*k*_ ≥ 0 and the diagonal entries in *D* are $D_{ii}=\sum_{j=1}^{n}A_{ij}$ for *i* ∈ {1, …, *n*}. Note that *L* = *Q*^⊺^ where *Q* is the standard generator matrix of the Markov process. It follows that, for any vector $x\in R^{n}$, *L* satisfies the following equation

$$\begin{array}{c} Lx\equiv \sum_{k=1}^{m}\zeta_{k}\alpha_{k}x_{i(k)}. \end{array}$$ (32)

The stoichiometry vector *ζ*_*k*_ is a difference of two standard unit vectors, *ζ*_*k*_ = *e*_*j*(*k*)_ − *e*_*i*(*k*)_. Although we do not assume that the graph Laplacian *L* must be a symmetric matrix, we do assume that the stationary system satisfies detailed balance, and that *L* has only real eigenvalues. Moreover, we assume that *L* has an expansion into real-valued biorthogonal eigentriples (*w*_λ_, λ, *v*_λ_) such that

$$Lv_{\lambda }=\lambda v_{\lambda }$$ (33)

$$L^{⊺}w_{\lambda }=\lambda w_{\lambda }$$ (34)

$$w_{\lambda }^{⊺}v_{\lambda^{\prime }}=\delta_{\lambda \lambda^{\prime }}.$$ (35)

We further assume that G is connected and the process is irreducible. The Perron-Frobenius theory guarantees the existence of a unique null eigenvector with nonnegative components summing to unity, corresponding to the stationary distribution on the graph. We denote the stationary probability vector $\vec{\pi }={\left(\pi_{1},\ldots ,\pi_{n}\right)}^{⊺}$ and the stationary mean flux along edge *k* by *J*_*k*_ = *N*_tot_*α*_*k*_*π*_*i*(*k*)_.

Let *B* be the *n* × *m* matrix defined such that

$$\begin{array}{c} B=(\begin{array}{cccc} \sqrt{J_{1}}\zeta_{1} & \sqrt{J_{2}}\zeta_{2} & \cdots & \sqrt{J_{m}}\zeta_{m} \end{array}). \end{array}$$ (36)

In other words, the *k*^th^ column of *B* is given by the square root of the stationary flux *J*_*k*_ multiplied by the stoichoimetry vector *ζ*_*k*_. We can express *B* as a sum of matrices

$$\begin{array}{c} B=\sum_{k=1}^{m}B_{k} \end{array}$$ (37)

where all the entries of *B*_*k*_ are zero except for the *k*^th^ column. Moreover, we will exploit the fact that the product *BB*^⊺^ can be represented with a similar sum

$$\begin{array}{c} BB^{⊺}=\sum_{k=1}^{m}B_{k}B_{k}^{⊺}. \end{array}$$ (38)

This product appears on the right hand side of the Lyapunov equation (see Eq 46 below) and its decomposition into the above sum is a key factor in establishing the decomposition of the stationary variance into a sum over the edges.

Computations were done either by hand, or using Matlab or Mathematica.

### Summary of stochastic shielding in the Langevin case

In the Langevin approximation for a time homogeneous first-order transition network, the population fraction occupying states 1, …, *n* is a vector $X\in R^{n}$ satisfying

$$\begin{array}{c} \frac{dX}{dt}=LX+\sum_{k\in E}B_{k}\xi_{k} \end{array}$$ (39)

where *L*, the graph Laplacian, captures the mean flux along each directed edge k∈E. The matrix *B*_*k*_ gives the effects of fluctuations *ξ*_*k*_ around the mean flux along the *k*^th^ edge. The noise terms are independent, white and Gaussian, with 〈*ξ*_*k*_(*t*)*ξ*_*k*′_(*t*′)〉 = *δ*_*kk*′_ *δ*(*t* − *t*′), one for each directed edge. Given an observable of interest, represented by a vector $M\in R^{n}$, the stochastic shielding approximation consists in finding a partition of the edge set, $E=E_{1}∐E_{2}$, into edges of *primary* importance ($E_{1}$) and *secondary* importance ($E_{2}$) such that $|E_{2}\left|\gg \right|E_{1}|$ and, at the same time $\lim_{t\to \infty }E||M^{⊺}{\left(Y\left(t\right)-X\left(t\right)\right)||}^{2}⪡\lim_{t\to \infty }E||M^{⊺}X\left(t\right){\left|\right|}^{2}$ (stationary variances), where **Y** is the approximate population vector satisfying

$$\begin{array}{c} \frac{dY}{dt}=LY+\sum_{k\in E_{1}}B_{k}\xi_{k},\;Y\left(0\right)=X\left(0\right). \end{array}$$ (40)

The noise samples *ξ*_*k*_ for $k\in E_{1}$ are identical in the full and approximate models. Neglecting the noise forcing along the edges of secondary importance causes a pathwise discrepancy **U**(*t*) = **Y**(*t*) − **X**(*t*) that satisfies

$$\begin{array}{c} \frac{dU}{dt}=LU-\sum_{k\in E_{2}}B_{k}\xi_{k},\;U\left(0\right)=0. \end{array}$$ (41)

The stochastic shielding *effect* consists in suppression of the resulting fluctuations in the observable process **M**^⊺^**U**(*t*) due to the filtering effects of the network—hence “stochastic shielding”.

The (stationary) mean squared pathwise approximation error can be written exactly as a sum of contributions *R*_*k*_ from each directed edge neglected in the approximation, $\lim_{t\to \infty }E||M^{⊺}{U\left(t\right))||}^{2}=\sum_{k\in E_{2}}R_{k}$. This error is small compared to $\lim_{t\to \infty }E||M^{⊺}{X\left(t\right))||}^{2}=\sum_{k\in E}R_{k}=\sum_{k\in E_{1}}R_{k}+\sum_{k\in E_{2}}R_{k}$. We call *R*_*k*_ the *importance* of the *k*^th^ directed edge (defined in the next section). As we show below, the decomposition holds exactly not only for the Langevin process but for the discrete population process as well.

### Edge importance measure

The general formula for the edge importance measure is as follows. For an arbitrary stationary population process **N**(*t*) satisfying detailed balance on a graph with *n* nodes, *m* edges, and measurement vector **M** (defining the observable states), $R=\sum_{k=1}^{m}R_{k}$ is the stationary variance of the observable states where

$$\begin{array}{c} R_{k}=J_{k}\sum_{i=2}^{n}\sum_{j=2}^{n}(\frac{-1}{\lambda_{i}+\lambda_{j}})\left(M^{⊺}v_{i}\right)\left(w_{i}^{⊺}\zeta_{k}\right)\left(\zeta_{k}^{⊺}w_{j}\right)\left(v_{j}^{⊺}M\right). \end{array}$$ (42)

In this formula, λ_*n*_ ≤ λ_*n*−1_ ≤ ⋯ ≤ λ_2_ < 0 are the nontrivial eigenvalues of the graph Laplacian *L* (which always has λ_1_ ≡ 0); *v*_*i*_ and *w*_*i*_ are the right and left eigenvectors of *L*, respectively. Here and henceforth, *R*_*k*_ is normalized to the variance due to a single random walker by dividing out *N*_tot_.

The stationary variance *R* is related to the power spectral density (PSD) *S*(*ω*) of the observed process **M**^⊺^**N**. From the Wiener-Khinchin theorem, integrating the PSD gives the stationary variance: $R=\int_{-\infty }^{\infty }S\left(\omega \right)\;d\omega$. Moreover, since the stationary variance decomposes into a sum of contributions from each edge in the graph, the power spectral density decomposes as well. By introducing

$$\begin{array}{c} R_{k}=\int_{-\infty }^{\infty }S_{k}\left(\omega \right)\;d\omega \end{array}$$ (43)

we define a *power-spectral edge importance* such that the integral of *S*_*k*_(*ω*), the power spectral density for the observed process with noise suppressed everywhere except edge *k*, gives the edge importance corresponding to edge *k*.

To see this, note that the power spectral density of the observed process is

$$\begin{array}{c} S\left(\omega \right)=\sum_{k\in E}S_{k}\left(\omega \right)\;\text{where} \end{array}$$ (44)

$$\begin{array}{c} S_{k}\left(\omega \right)=\frac{1}{2\pi }J_{k}\sum_{l=2}^{n}\sum_{j=2}^{n}(\frac{1}{\lambda_{l}+i\omega })(\frac{1}{\lambda_{j}-i\omega })(M^{⊺}v_{l})(u_{l}^{⊺}\zeta_{k})(\zeta_{k}^{⊺}u_{j})(v_{j}^{⊺}M) \end{array}$$ (45)

provided *ω* > 0. For more details, see §Numerical Methods: Calculation of power spectra, below. We can use this power spectral decomposition to explore how the spectral contributions differ between the typical cases (where edge importance ranking agrees with the stochastic shielding method) and in the edge importance reversal cases.

### Lyapunov equation

The Perron-Frobenius null eigenvector, suitably normalized, gives the stationary probability vector $\vec{\pi }={\left(\pi_{1},\ldots ,\pi_{n}\right)}^{⊺}$ of Markov process **N**(*t*). Snapshots of the process **N**(*t*), taken under stationary conditions, are multinomial with parameters $N_{\text{tot}},\vec{\pi }$, so the covariance matrix *C* is known. In particular, each diagonal entry in *C* is the variance of state *i*, *C*_*ii*_ = *N*_tot_*π*_*i*_(1 − *π*_*i*_), and each off-diagonal entry in *C* is the covariance of states *i* and *j*, *C*_*ij*_ = −*N*_tot_*π*_*i*_*π*_*j*_ for *i* ≠ *j*.

The stationary covariance matrix *C* satisfies Lyapunov’s equation (a special case of Sylvester’s equation) [43]

$$\begin{array}{c} LC+CL^{⊺}=-BB^{⊺}. \end{array}$$ (46)

The fact that *C* satisfies Eq 46 above is widely known for linear Gaussian processes such as multivariate Ornstein-Uhlenbeck processes [34], but it also holds for discrete state population processes in which the transition rates are linear functions of the population at each node, *i.e.* first-order transition networks, such as those we consider here (see [8, 44]).

Our system is an important special case of the general first-order reaction network presented in [8]; we only consider conversion type reactions (denoted by *k*^con^ in [8]). For our system *P*_*i*_ represents $v_{\lambda }u_{\lambda }^{⊺}$, summed over all identical λ if they occur with multiplicity (we both assume semisimple eigenvalue spectra). The following parameters in [8] are zero for our system: *C*(*i*, *k*, *l*), *k*^cat^, *k*^s^, and *k*^d^. This simplifies Equation 50 in [8] (representing the variance of the *l*^*th*^ reactant in the network) and is equivalent to our edge importance measure (Eq 42). However, to our knowledge, we are the first to describe the unique decomposition of the stationary variance into a sum of contributions from each edge in the network, and [9, 10] were the first to propose the stochastic shielding approximation and justify it based on this decomposition.

The Lyapunov equation has also been used in the context of stochastic gene networks under the name of “linear noise approximation” [45, 46]; in particular [45] (pg. 1, ¶5) further cites Eqs 3.46 and 6.115 in Risken [47] for additional details. See also [48] Supporting Information §4. For the linear networks we consider here, the equation is exact.

### Proof of Lemma 1

We restate the lemma for the reader’s convenience. Recall from Definition 1 that *Y* is the space of *n* × *n* symmetric matrices with columns (and rows) summing to zero.

**Lemma 1 (restated)** *Let L be an n* × *n real valued matrix with L*_*ij*_ ≥ 0 *for i* ≠ *j, and L*_*ii*_ = −∑_*i*,*i* ≠ *j*_ *L*_*ij*_ (*so that* $\sum_{i=1}^{n}L_{ij}=0$) *for j* = 1, …, *n, and satisfying dim*(*ker*(*L*)) = 1, *and with a null eigenvector Lv* = 0 *satisfying v*_*i*_ ≥ 0 *for i* = 1, …, *n. Then for any F* ∈ *Y, the equation*

$$LC+CL^{⊺}=F$$

*has a unique solution C* ∈ *Y*.

**Proof 2** *Proof of Lemma 1. Given L* ∈ *X, define the linear operator A by A*: *C* → *LC* + *CL*^⊺^. *First, we show that A*: *Y* → *Y. If C* ∈ *Y then for all j* = 1, …, *n*,

$$\sum_{i=1}^{n}{\left(LC+CL^{⊺}\right)}_{ij}=\sum_{i,k=1}^{n}\left(L_{ik}C_{kj}+C_{ik}L_{jk}\right)=\sum_{k=1}^{n}C_{kj}\sum_{i=1}^{n}L_{ik}+\sum_{k=1}^{n}L_{jk}\sum_{i=1}^{n}C_{ik}=0,$$ (47)

*because each sum over i is zero, by assumption. Moreover*, (*LC* + *CL*^⊺^)^⊺^ = *LC* + *CL*^⊺^. *Therefore LC* + *CL*^⊺^ ∈ *Y whenever C* ∈ *Y, so A maps Y into itself*.

*By the Fredholm alternative(cf. [49], Theorem 2.27), A(C) = F has a unique inverse for F* ∈ *Y provided F is in the range of A and the homogeneous equation A(C*) = 0 *has only the trivial solution C* = 0.

*Let C*_0_ ∈ *Y be a solution of the homogeneous equation*, *LC*_0_ + *C*_0_*L*^⊺^ = 0. *Because C*_0_ ∈ *Y is symmetric and the nullspace of L is one dimensional, C*_0_ *must have the form C*_0_ = (*c*_1_*v*|⋯|*c*_*n*_*v) for constants c*_1_, …, *c*_*n*_. *However, the columns of C*_0_ *must sum to zero, and* $\sum_{i=1}^{n}v_{i}>0$, *therefore c*_1_ = … = *c*_*n*_ = 0, *hence C*_0_ = 0.

*To see that F is in the range of A, we construct an explicit solution as follows*:

$$\begin{array}{c} C=\int_{0}^{\infty }e^{tL}F{\left(e^{tL}\right)}^{⊺}dt, \end{array}$$ (48)

*and we show that this integral is well defined whenever F* ∈ *Y. To see this, first note that if all eigenvalues of L have negative real part, then*

$$\begin{array}{c} LC+CL^{⊺}=\int_{0}^{\infty }Sdt \end{array}$$ (49)

*where*

$$S=Le^{tL}Fe^{tL^{⊺}}+e^{tL}Fe^{tL^{⊺}}L^{⊺}$$ (50)

$$=\frac{d}{dt}(e^{tL}Fe^{tL^{⊺}})$$ (51)

*and the solution in* Eq 48 *follows from the fundamental theorem of calculus*.

*It remains to show that the integral in* Eq 48 *is well defined whenever F* ∈ *Y. Assuming detailed balance, a unique null space, and that L is diagonalizable, we have that all eigenvalues of L are negative (and real) except* λ_1_ ≡ 0, *and we can write*

$$L=\sum_{\lambda }v_{\lambda }u_{\lambda }^{⊺}$$ (52)

$$\Rightarrow e^{tL}=v_{0}u_{0}^{⊺}+\sum_{\lambda <0}e^{t\lambda }v_{\lambda }u_{\lambda }^{⊺}.$$ (53)

*Then*

$$C=\int_{0}^{\infty }e^{tL}F{\left(e^{tL}\right)}^{⊺}dt$$ (54)

$$\begin{array}{cc} & =\int_{0}^{\infty }\{\left(v_{1}u_{1}^{⊺}\right)F{\left(v_{1}u_{1}^{⊺}\right)}^{⊺}+\left(v_{1}u_{1}^{⊺}\right)F{\left(\sum_{\lambda <0}e^{t\lambda }v_{\lambda }u_{\lambda }^{⊺}\right)}^{⊺} \end{array}$$ (55)

$$+\left(\underset{\lambda <0}{\sum }e^{t\lambda }v_{\lambda }u_{\lambda }^{⊺}\right)F{\left(v_{1}u_{1}^{⊺}\right)}^{⊺}+\underset{\lambda <0,\lambda^{\prime }<0}{\sum }e^{t\left(\lambda +\lambda^{\prime }\right)}v_{\lambda }u_{\lambda }^{⊺}Fu_{\lambda^{\prime }}v_{\lambda^{\prime }}^{⊺}\}dt$$ (56)

$$\begin{array}{cc} & =\int_{0}^{\infty }\{v_{1}\left(\underline{u_{1}^{⊺}F}\right)\left(u_{1}v_{1}^{⊺}\right)+v_{1}\left(\underline{u_{1}^{⊺}F}\right)\sum_{\lambda <0}e^{t\lambda }u_{\lambda }v_{\lambda }^{⊺} \end{array}$$ (57)

$$\begin{array}{cc} & \;+\sum_{\lambda <0}e^{t\lambda }v_{\lambda }u_{\lambda }^{⊺}\left(\underline{Fu_{1}}\right)v_{1}^{⊺}+\sum_{\lambda <0,\lambda^{\prime }<0}e^{t\left(\lambda +\lambda^{\prime }\right)}v_{\lambda }u_{\lambda }^{⊺}Fu_{\lambda^{\prime }}v_{\lambda^{\prime }}^{⊺}\}dt \end{array}$$ (58)

$$\begin{array}{cc} & =\sum_{\lambda <0,\lambda^{\prime }<0}v_{\lambda }u_{\lambda }^{⊺}Fu_{\lambda^{\prime }}v_{\lambda^{\prime }}^{⊺}(\int_{0}^{\infty }e^{t(\lambda +\lambda^{\prime })}\;dt) \end{array}$$ (59)

$$\begin{array}{cc} & =\sum_{\lambda <0,\lambda^{\prime }<0}\frac{-1}{\lambda +\lambda^{\prime }}v_{\lambda }u_{\lambda }^{⊺}Fu_{\lambda^{\prime }}v_{\lambda^{\prime }}^{⊺}. \end{array}$$ (60)

*The underlined expressions in parentheses are all zero because the columns (and rows since F is a symmetric matrix) of F sum to zero by assumption*; $u_{1}^{⊺}\equiv \left(1,\ldots ,1\right)$ *is orthogonal to every column of F and u*_1_ *is orthogonal to every row of F and so* $u_{1}^{⊺}F=0$ *and Fu*_1_ = 0. *Thus, the integral in* Eq 54 *is finite and* Eq 60 *gives an explicit expression for it*.

### Numerical methods

#### Discrete state simulations

To represent the trajectory of the state or the measurement functional (*e.g.* ion channel conductance) due to a single random walker, as for instance in Fig 2, we used Gillespie’s exact stochastic simulation algorithm (SSA) [50] implemented in Matlab. Briefly, the SSA is a method for constructing simulated trajectories of finite populations in continuous time. If *N*_*i*_(*t*) is the number of individuals in state *i* (for *i* ∈ {1, …, *n*}) at time *t*, the SSA generates the state vector **N**(*t*) = (*N*_1_(*t*), …, *N*_*n*_(*t*)) given that the system was initially in state **N**(*t*_0_) = *x*_0_ at time *t*_0_. Reactions cause the state of the system to change over time. The SSA method samples the time *τ* to the next reaction and updates the state of the system accordingly.

#### Continuous state simulations

To represent the trajectory of a population (*N*_tot_ = 500) of random walkers for the full processes or different fluctuation-suppressed approximations derived from the stochastic shielding method, as for instance in Figs 4 and 8–10, we used a Langevin approximation. Briefly, we consider a linear Langevin equation with strictly additive noise, given by

$$\begin{array}{c} dX=LX\;dt+B\;dW \end{array}$$ (61)

where $X\left(t\right)\approx N\left(t\right)-\bar{N}$ and the graph Laplacian *L* and matrix *B* are those considered in Eqs 32–36. See S1 Supporting Information §Connection to Gaussian approximation for more details, and note that Eq S40–S42 define *L* and *B* specifically for the 3-state process. We used the Euler-Maruyama method implemented in Matlab to numerically solve the SDE above.

#### Calculation of power spectra

See Equation 4.5.78 in Gardiner §4.5.6 for the spectrum matrix of a stationary multivariate Ornstein-Uhlenbeck process [34]. The power spectrum of the observable process **M**^⊺^**X** is

$$S\left(\omega \right)=\frac{1}{2\pi }M^{⊺}{\left(L+i\omega \right)}^{-1}BB^{⊺}{\left(L^{⊺}-i\omega \right)}^{-1}M$$ (62)

$$=\frac{1}{2\pi }\sum_{\lambda }\sum_{\lambda^{\prime }}(\frac{1}{\lambda +i\omega })(\frac{1}{\lambda^{\prime }-i\omega })\sum_{k\in E}J_{k}(M^{⊺}v_{\lambda })(u_{\lambda }^{⊺}\zeta_{k})(\zeta_{k}^{⊺}u_{\lambda^{\prime }})(v_{\lambda^{\prime }}^{⊺}M)$$ (63)

$$=\frac{1}{2\pi }\sum_{k\in E}J_{k}\sum_{\lambda }\sum_{\lambda^{\prime }}(\frac{1}{\lambda +i\omega })(\frac{1}{\lambda^{\prime }-i\omega })(M^{⊺}v_{\lambda })(u_{\lambda }^{⊺}\zeta_{k})(\zeta_{k}^{⊺}u_{\lambda^{\prime }})(v_{\lambda^{\prime }}^{⊺}M)$$ (64)

$$=\sum_{k\in E}S_{k}\left(\omega \right),\;\text{where}\;$$ (65)

$$S_{k}\left(\omega \right)=\frac{1}{2\pi }J_{k}\sum_{\lambda }\sum_{\lambda^{\prime }}(\frac{1}{\lambda +i\omega })(\frac{1}{\lambda^{\prime }-i\omega })(M^{⊺}v_{\lambda })(u_{\lambda }^{⊺}\zeta_{k})(\zeta_{k}^{⊺}u_{\lambda^{\prime }})(v_{\lambda^{\prime }}^{⊺}M)$$ (66)

provided *ω* > 0. The left eigenvector for λ = 0 is orthogonal to *ζ*_*k*_ for each edge *k*, so the sum *de facto* excludes all terms with λ = 0 or λ′ = 0. Notice that the integral of the power spectrum corresponding to edge *k* gives the edge importance for edge *k*:

$$\begin{array}{c} R_{k}=\int_{-\infty }^{\infty }S_{k}\left(\omega \right)\;d\omega . \end{array}$$ (67)

More generally, one could use the lagged covariance of the Gaussian process, given by Equation 4.5.71 in Gardiner §4.5.6 [34]

$$\begin{array}{cc} \left\langle x\left(t\right),x^{⊺}\left(s\right)\right\rangle & =\exp\left(Lt\right)\left\langle x\left(0\right),x^{⊺}\left(0\right)\right\rangle \exp\left(L^{⊺}s\right)\\ & \;+\int_{0}^{s\wedge t}\exp[L(t-t^{\prime })]BB^{⊺}\exp[L^{⊺}\left(s-t^{\prime }\right)]dt^{\prime }. \end{array}$$ (68)

If we write instead (assuming *s* = *t* + *τ*, *τ* ≥ 0)

$$\begin{array}{ll} \langle x(t),x^{⊺}(t+\tau )\rangle & =\text{exp}(Lt)\langle x(a),x^{⊺}(a)\rangle \text{exp}(L^{⊺}\tau )\\ & +\int_{a}^{t}\text{exp}\left[L(t-t^{\prime })\right]BB^{⊺}\text{exp}\left[L^{⊺}(t+\tau -t^{\prime })\right]dt^{\prime }, \end{array}$$ (69)

and take the limit *a* → −∞, then we see that *C*(*τ*), the stationary lagged covariance at lag *τ*, satisfies *C*(*τ*) = ∑_*k*_ *C*_*k*_(*τ*), where

$$\begin{array}{cc} C_{k}\left(\tau \right) & =\sum_{\lambda ,\lambda^{\prime }<0}\frac{-e^{\lambda \tau }}{\lambda +\lambda^{\prime }}v_{\lambda }u_{\lambda }^{⊺}B_{k}B_{k}^{⊺}u_{\lambda^{\prime }}v_{\lambda^{\prime }}^{⊺}. \end{array}$$ (70)

Multiplying by the measurement vector *M* and taking the Fourier transform of this expression yields *S*_*k*_(*ω*) above.

## Supporting information

S1 Supporting Information We establish the decomposition of the stationary variance and calculate *η*, the fraction of variance of the observable state arising from the hidden edges, providing explicit calculations for the 3-state process. We also detail the connection between the population process and Gaussian approximations thereof, and derive the Lyapunov equation for the 3-state case.
(PDF)
