# Supplementary material for: Stochastic shielding and edge importance for Markov chains with timescale separation
Source: PLoS Comput Biol. 2018 Jun 18;14(6):e1006206. doi: 10.1371/journal.pcbi.1006206 (PMC6023243; doi:10.1371/journal.pcbi.1006206)
Supplement: S1 Supporting Information — We establish the decomposition of the stationary variance and calculate η, the fraction of variance of the observable state arising from the hidden edges, providing explicit calculations for the 3-state process. We also detail the connection between the population process and Gaussian approximations thereof, and derive the Lyapunov equation for the 3-state case. (PDF) [file pcbi.1006206.s001.pdf]

# S1 Supporting Information

We establish the decomposition of the stationary variance and calculate $\eta$, the fraction of variance of the observable state arising from the hidden edges, providing explicit calculations for the 3-state process. We also review the connection between the population process and Gaussian approximations thereof, and give a detailed derivation of the Lyapunov equation for the 3-state case.

We show in Lemma 1 and Theorem 1 (see Results) that there is a unique decomposition of the stationary covariance into a sum of contributions from each edge in the graph (or, in the case of detailed balance, each reciprocal pair of edges). Since the stationary covariance matrix $C$ satisfies the Lyapunov equation (Eq 46) and $BB^\intercal$ decomposes into a sum of contributions from each edge, we can establish the linear decomposition of $C$ as sum of contributions from each edge. This allows us to rank each edge in terms of its importance, and it allows us to derive the accuracy of the stochastic shielding approximation. This result holds for the exact underlying process $\mathbf{N}(t)$ and for any number of particles $N_{\text{tot}} \geq 1$; the result is not based on an approximation. We derive this decomposition for the 3-state process in following section. To our knowledge, this decomposition has not been shown previously.

## Decomposition of the Stationary Variance: 3-State Process

This section gives a detailed description of the framework and derivation of results for the 3-state process. We also make the connection between our previous work, a Gaussian approximation of the exact process [10], and the results for the exact discrete-state formulation in the current paper.

The exact formulation of the 3-state process is a discrete state, continuous time Markov jump process $\mathbf{N}(t)$ (see Fig 3 for an illustration of the transition state graph, and note that state 3 is the observable state which means that the measurement vector in this case is $M = \begin{pmatrix} 0 & 0 & 1 \end{pmatrix}^\intercal$). Specifically, $\mathbf{N}(t)$ is an integer-valued process that can be described using the random time change representation [40, 41] in terms of four independent unit rate Poisson processes $Y_1, Y_2, Y_3, Y_4$:

$$\mathbf{N}(t) = \mathbf{N}(0) + \sum_{k=1}^{4} \zeta_k Y_k \left[ \int_0^t N_{i(k)}(s) \alpha_k \, ds \right]. \tag{S1}$$

Here, $\mathbf{N}(t) = (N_1(t), N_2(t), N_3(t))^\intercal$ is the state occupancy vector at time $t$ and satisfies $\sum_{i=1}^{3} N_i(t) \equiv N_{\text{tot}}$. The stoichiometry vectors $\zeta_k$ are given by

$$\zeta_1 = \begin{pmatrix} -1 \\ 1 \\ 0 \end{pmatrix}, \qquad \zeta_2 = \begin{pmatrix} 1 \\ -1 \\ 0 \end{pmatrix}, \qquad \zeta_3 = \begin{pmatrix} 0 \\ -1 \\ 1 \end{pmatrix}, \qquad \zeta_4 = \begin{pmatrix} 0 \\ 1 \\ -1 \end{pmatrix} \tag{S2}$$

where edge $k$ represents a transition from source node $i(k)$ to destination node $j(k)$. The graph Laplacian $L$ and matrix $B$ for the 3-state process are given by

$$L = \begin{pmatrix} -\alpha_1 & \alpha_2 & 0 \\ \alpha_1 & -\alpha_2 - \alpha_3 & \alpha_4 \\ 0 & \alpha_3 & -\alpha_4 \end{pmatrix}, \quad B = \begin{pmatrix} -\sqrt{J_1} & \sqrt{J_2} & 0 & 0 \\ \sqrt{J_1} & -\sqrt{J_2} & -\sqrt{J_3} & \sqrt{J_4} \\ 0 & 0 & \sqrt{J_3} & -\sqrt{J_4} \end{pmatrix}, \tag{S3}$$

where $J_k = N_{\text{tot}} \alpha_k \pi_{i(k)}$ is the stationary flux along the $k^{\text{th}}$ edge (computed below). The steady state node occupancy probabilities $\pi_i$ for the 3-state process are

$$\pi_1 = \alpha_2\alpha_4/(\alpha_1\alpha_3 + \alpha_1\alpha_4 + \alpha_2\alpha_4) \tag{S4}$$

$$\pi_2 = \alpha_1\alpha_4/(\alpha_1\alpha_3 + \alpha_1\alpha_4 + \alpha_2\alpha_4) \tag{S5}$$

$$\pi_3 = \alpha_1\alpha_3/(\alpha_1\alpha_3 + \alpha_1\alpha_4 + \alpha_2\alpha_4). \tag{S6}$$

Detailed balance is easy to establish. The steady state flux $J_k$ along edge $k$ is

$$J_1 = \pi_1\alpha_1 = \frac{\alpha_1\alpha_2\alpha_4}{\alpha_1\alpha_3 + \alpha_1\alpha_4 + \alpha_2\alpha_4} = \pi_2\alpha_2 = J_2 \tag{S7}$$

$$J_3 = \pi_2\alpha_3 = \frac{\alpha_1\alpha_3\alpha_4}{\alpha_1\alpha_3 + \alpha_1\alpha_4 + \alpha_2\alpha_4} = \pi_3\alpha_4 = J_4. \tag{S8}$$

Note that the stationary distribution for the $N_{\text{tot}}$ random walkers is the multinomial distribution with probability vector $\vec{\pi} = (\pi_1, \pi_2, \pi_3)^\intercal$. The stationary mean is $\bar{\mathbf{N}} = E[\mathbf{N}] = N_{\text{tot}}\vec{\pi}$. The stationary covariance is $C = \text{diag}[\vec{\pi}] - \vec{\pi}\vec{\pi}^\intercal$, *i.e.* the variance of the population at node $i$ is $N_{\text{tot}}\pi_i(1-\pi_i)$ and the covariance of nodes $i$ and $j$ is $-N_{\text{tot}}\pi_i\pi_j$.

If we let $R = C_{3,3}$ be the stationary variance of state 3, then we have

$$R = \sum_{k=1}^{4} R_k \tag{S9}$$

where

$$R_k = J_k \sum_{i=2}^{3}\sum_{j=2}^{3} \left(\frac{-1}{\lambda_i + \lambda_j}\right)(M^\intercal v_i)(w_i^\intercal \zeta_k)(\zeta_k^\intercal w_j)(v_j^\intercal M). \tag{S10}$$

Note that this is a special case of Eq 42. In this formula, $J_k$ is the stationary flux along the $k^{th}$ edge, and $\lambda_3 \le \lambda_2 < 0$ are the two nontrivial eigenvalues of the graph Laplacian $L$ (which always has $\lambda_1 \equiv 0$). The corresponding right and left eigenvectors are $v_3, v_2$ and $w_3^\intercal, w_2^\intercal$, respectively. Along with $v_1 = \vec{\pi}$ and $w_1 = (1,1,1)^\intercal$, the eigenvectors are normalized to form a biorthogonal set, i.e. $w_i^\intercal v_j = \delta_{ij}$.

We can obtain the eigenvectors and eigenvalues explicitly for each of the twelve cases of the 3-state chain as a function of the parameter $\alpha$ (See Table 3 for a description of the cases; see the next section for eigenvectors and eigenvalues). Then we can examine the range of applicability of the stochastic shielding method [9], the edge importance measure [10], and the connection to the power spectrum.

## Explicit calculation of $\eta$ (fraction of variance generated by the hidden edges)

To calculate $\eta \equiv \dfrac{R_{12}}{R_{12} + R_{23}}$, the fraction of the stationary variance of the observable state coming from the hidden edges in the 3-state chain, we derive expressions for the eigenvalues and eigenvectors of the graph Laplacian $L$ as functions of the transition rates $\alpha_{ij}$. The eigenvalues are given by

$$\lambda_1 = 0, \tag{S11}$$

$$\lambda_2 = \frac{1}{2}\left(-\sqrt{\alpha_{\text{sum}}^2 - 4Z} - \alpha_{\text{sum}}\right), \tag{S12}$$

$$\lambda_3 = \frac{1}{2}\left(\sqrt{\alpha_{\text{sum}}^2 - 4Z} - \alpha_{\text{sum}}\right). \tag{S13}$$

where $\alpha_{\text{sum}} = \alpha_{12} + \alpha_{21} + \alpha_{23} + \alpha_{32}$ and $Z = \alpha_{12}\alpha_{23} + \alpha_{12}\alpha_{32} + \alpha_{21}\alpha_{32}$. Notice that $\alpha_{\text{sum}} = -\text{Tr}[L]$ where $\text{Tr}[L]$ is the trace of $L$. The right eigenvectors are

$$v_1 = \left\{\frac{\alpha_{21}\alpha_{32}}{Z}, \frac{\alpha_{12}\alpha_{32}}{Z}, \frac{\alpha_{12}\alpha_{23}}{Z}\right\} \tag{S14}$$

$$v_2 = \left\{\sqrt{\alpha_{\text{sum}}^2 - 4Z} + \alpha_{12} + \alpha_{21} - \alpha_{23} - \alpha_{32}, -\sqrt{\alpha_{\text{sum}}^2 - 4Z} + \alpha_{\text{sum}} + 2\alpha_{32}, 2\alpha_{23}\right\} \tag{S15}$$

$$v_3 = \left\{-\sqrt{\alpha_{\text{sum}}^2 - 4Z} + \alpha_{12} + \alpha_{21} - \alpha_{23} - \alpha_{32}, \sqrt{\alpha_{\text{sum}}^2 - 4Z} + \alpha_{\text{sum}} + 2\alpha_{32}, 2\alpha_{23}\right\} \tag{S16}$$

The left eigenvectors are

$$u_1 = \{1, 1, 1\} \tag{S17}$$

$$u_2 = \{u_2^{(1)}, u_2^{(2)}, u_2^{(3)}\} \tag{S18}$$

$$u_3 = \{u_3^{(1)}, u_3^{(2)}, u_3^{(3)}\} \tag{S19}$$

where

$$u_2^{(1)} = \frac{\alpha_{12}\left(-\sqrt{\alpha_{\text{sum}}^2 - 4Z} + \alpha_{\text{sum}}\right)}{4Z\sqrt{\alpha_{\text{sum}}^2 - 4Z}} \tag{S20}$$

$$u_2^{(2)} = \frac{\alpha_{12}^2 + \alpha_{12}(\alpha_{21} - \alpha_{23} - \alpha_{32}) - \alpha_{12}\sqrt{\alpha_{\text{sum}}^2 - 4Z} - 2\alpha_{21}\alpha_{32}}{4Z\sqrt{\alpha_{\text{sum}}^2 - 4Z}} \tag{S21}$$

$$u_2^{(3)} = \frac{2\alpha_{21}\alpha_{32}}{\alpha_{12}W_1^2 + \alpha_{21}W_2^2 + 4\alpha_{21}\alpha_{23}\alpha_{32}} \tag{S22}$$

where $W_1 = \sqrt{\alpha_{\text{sum}}^2 - 4Z} + \alpha_{12} + \alpha_{21} - \alpha_{23} - \alpha_{32}$, $W_2 = \sqrt{\alpha_{\text{sum}}^2 - 4Z} + \alpha_{\text{sum}} - 2\alpha_{32}$, and

$$u_3^{(1)} = -\frac{\alpha_{12}\left(\sqrt{\alpha_{\text{sum}}^2 - 4Z} + \alpha_{\text{sum}}\right)}{4Z\sqrt{\alpha_{\text{sum}}^2 - 4Z}} \tag{S23}$$

$$u_3^{(2)} = \frac{-\alpha_{12}^2 - \alpha_{12}(\alpha_{21} - \alpha_{23} - \alpha_{32}) - \alpha_{12}\sqrt{\alpha_{\text{sum}}^2 - 4Z} + 2\alpha_{21}\alpha_{32}}{4Z\sqrt{\alpha_{\text{sum}}^2 - 4Z}} \tag{S24}$$

$$u_3^{(3)} = \frac{2\alpha_{21}\alpha_{32}}{\alpha_{12}(W_1')^2 + \alpha_{21}(W_2')^2 + 4\alpha_{21}\alpha_{23}\alpha_{32}} \tag{S25}$$

where $W_1' = \sqrt{\alpha_{\text{sum}}^2 - 4Z} - \alpha_{12} - \alpha_{21} + \alpha_{23} + \alpha_{32}$, $W_2' = -\sqrt{\alpha_{\text{sum}}^2 - 4Z} + \alpha_{\text{sum}} - 2\alpha_{32}$.

The variance components are given by

$$R_{12} = \frac{\alpha_{12}\alpha_{21}\alpha_{23}^2\alpha_{32}}{2\alpha_{\text{sum}}Z^2}$$

$$R_{23} = \frac{\alpha_{12}\alpha_{23}\alpha_{32}\left(\alpha_{12}^2 + \alpha_{21}(\alpha_{21} + \alpha_{32}) + \alpha_{12}(2\alpha_{21} + \alpha_{23} + \alpha_{32})\right)}{2\alpha_{\text{sum}}Z^2}$$

which correspond to the contributions made by the hidden and visible edges, respectively. Note that $R_{12} = R_{21}$ and $R_{23} = R_{32}$ due to detailed balance. The total variance is $V_{\text{tot}} = 2(R_{12} + R_{23})$:

$$V_{\text{tot}} = \frac{\alpha_{12}\alpha_{23}\alpha_{32}(\alpha_{12} + \alpha_{21})}{Z^2}.$$

It follows that (verified by Mathematica and simplifying terms by hand)

$$\eta \equiv \frac{R_{12}}{R_{12} + R_{23}} = \left(\frac{\alpha_{21}}{\alpha_{12} + \alpha_{21}}\right)\left(\frac{\alpha_{23}}{\alpha_{12} + \alpha_{21} + \alpha_{23} + \alpha_{32}}\right) \tag{S26}$$

$$= \left(\frac{\pi_1}{\pi_1 + \pi_2}\right)\left(\frac{\alpha_{23}}{\text{Tr}[-L]}\right). \tag{S27}$$

The set of equations above are equivalent to Eq 22 given in Results (note that the equality of the first factors in Eq S26 and S27 can be easily derived from Eq S4 - S6). Edge importance is reversed when $R_{12} > R_{23}$, and this corresponds to the condition $\eta > 1/2$.

### Canonical transformation leading to edge importance reversal: detailed calculations

On page 16 of the main paper, we introduced a canonical transformation of a given set of transition rates that lead to edge importance reversal. Here we provide the details justifying our assertions. Given an arbitrary initial set of transition rates $\alpha_{ij}^0 > 0$ and $\epsilon > 0$, define the following rescaled transition rates

$$\alpha_{12} = \epsilon\alpha_{12}^0 \quad \text{(decelerate transition } 1 \to 2\text{)} \tag{S28}$$

$$\alpha_{21} = \alpha_{21}^0 \tag{S29}$$

$$\alpha_{23} = \frac{1}{\epsilon}\alpha_{23}^0 \quad \text{(accelerate transition } 2 \to 3\text{)} \tag{S30}$$

$$\alpha_{32} = \alpha_{32}^0. \tag{S31}$$

We will show that as $\epsilon$ approaches 0, we eventually reverse the edge importance and then approach $\eta = R_{12}/(R_{12}+R_{23}) \to 1$. At the same time $\pi_2 \to 0$, the timescale separation grows, as does the flux imbalance.

The stationary probabilities satisfy

$$\pi_1 = \alpha^0_{32}\alpha^0_{21}/Z \sim O(1), \text{ as } \epsilon \to 0^+ \tag{S32}$$
$$\pi_2 = \epsilon\alpha^0_{32}\alpha^0_{12}/Z \sim O(\epsilon), \text{ as } \epsilon \to 0^+ \tag{S33}$$
$$\pi_3 = \alpha^0_{12}\alpha^0_{23}/Z \sim O(1), \text{ as } \epsilon \to 0^+ \tag{S34}$$
$$Z = \alpha^0_{32}\alpha^0_{21} + \epsilon\alpha^0_{32}\alpha^0_{12} + \alpha^0_{12}\alpha^0_{23} \sim O(1), \text{ as } \epsilon \to 0^+, \tag{S35}$$

so for any choice of $\alpha^0_{ij} > 0$, $\pi_2$ becomes arbitrarily small for sufficiently small $\epsilon$.

Similarly, it is easy to see that $J_{12} = \pi_1\alpha_{12} = \epsilon\alpha^0_{12}\alpha^0_{21}\alpha^0_{32}/Z \sim O(\epsilon)$, while $J_{23} = \pi_2\alpha_{23} = \alpha^0_{12}\alpha^0_{23}\alpha^0_{32}/Z \sim O(1)$, as $\epsilon \to 0^+$. So the ratio $J_{12}/J_{23} = \epsilon\alpha^0_{21}/\alpha^0_{23} \sim O(\epsilon)$ as $\epsilon$ decreases. Thus, the condition that the majority of the stationary flux occurs along the observable edge is guaranteed to hold for sufficiently small $\epsilon$.

In addition, as $\epsilon$ decreases the timescale separation grows without bound. The timescale factor $\nu = \lambda_3/\lambda_2$ (the ratio of the two non-zero eigenvalues) grows quadratically with $\epsilon$:

$$\nu = \left(\frac{\alpha_{12}\alpha_{23} + \alpha_{21}\alpha_{32}}{\alpha^2_{23}}\right)\epsilon^2 + O(\epsilon^3), \text{ as } \epsilon \to 0^+. \tag{S36}$$

The fraction of the stationary variance due to the hidden edges satisfies

$$\eta = 1 - \left(\frac{\alpha^2_{21} + \alpha_{12}\alpha_{23} + \alpha_{32}\alpha_{21}}{\alpha_{21}\alpha_{23}}\right)\epsilon + O(\epsilon^2), \text{ as } \epsilon \to 0^+. \tag{S37}$$

Thus, for any positive values of $\alpha^0_{ij}$, accelerating the $2 \to 3$ transition while decelerating the $2 \to 1$ transition will eventually lead to strongly inverted edge importance, with an arbitrarily large fraction of the stationary variance in the occupancy of the third state arising from the fluctuations in the transitions between nodes 1 and 2.

## Connection to Gaussian approximation

We now facilitate going back and forth between the two formulations (Gaussian approximation and exact formulation) as we spell out the connection to our previous work [10]. We illustrate this for the 3-state process, but the connection holds in general.

For a given set of fixed transition rates $\alpha_k$, and sufficiently large population size $N_{\text{tot}}$, we may approximate the integer process $\mathbf{N}(t)$ with the solution of a Langevin equation with independent Gaussian white noise forcing arising from each directed edge [42], §8. Namely, we have the Itô stochastic differential equation

$$d\mathbf{X} = L\,\mathbf{X}\,dt + B(\mathbf{X})\,d\mathbf{W} \tag{S38}$$

with linear drift $L\,\mathbf{X}$ and state dependent noise amplitude $B(\mathbf{X})$ given by the matrices

$$L = \begin{pmatrix} -\alpha_1 & \alpha_2 & 0 \\ \alpha_1 & -\alpha_2-\alpha_3 & \alpha_4 \\ 0 & \alpha_3 & -\alpha_4 \end{pmatrix}, \quad B = \begin{pmatrix} -\sqrt{\alpha_1 X_1} & \sqrt{\alpha_2 X_2} & 0 & 0 \\ \sqrt{\alpha_1 X_1} & -\sqrt{\alpha_2 X_2} & -\sqrt{\alpha_3 X_2} & \sqrt{\alpha_4 X_3} \\ 0 & 0 & \sqrt{\alpha_3 X_2} & -\sqrt{\alpha_4 X_3} \end{pmatrix}. \tag{S39}$$

As a further simplification, valid for yet larger $N_{\text{tot}}$, we may consider a linear Langevin equation with strictly additive noise, given by

$$d\tilde{\mathbf{X}} = L\tilde{\mathbf{X}}\,dt + B_0\,d\mathbf{W} \tag{S40}$$

with $L$ as above, and

$$B_0 = \begin{pmatrix} -\sqrt{\alpha_1\bar{N}_1} & \sqrt{\alpha_2\bar{N}_2} & 0 & 0 \\ \sqrt{\alpha_1\bar{N}_1} & -\sqrt{\alpha_2\bar{N}_2} & -\sqrt{\alpha_3\bar{N}_2} & \sqrt{\alpha_4\bar{N}_3} \\ 0 & 0 & \sqrt{\alpha_3\bar{N}_2} & -\sqrt{\alpha_4\bar{N}_3} \end{pmatrix} \quad \text{(S41)}$$

$$= \begin{pmatrix} -\sqrt{J_1} & \sqrt{J_2} & 0 & 0 \\ \sqrt{J_1} & -\sqrt{J_2} & -\sqrt{J_3} & \sqrt{J_4} \\ 0 & 0 & \sqrt{J_3} & -\sqrt{J_4} \end{pmatrix} \quad \text{(S42)}$$

where matrix $B_0$ depends on the equilibrium population size $\bar{\mathbf{N}} = [\bar{N}_1, \bar{N}_2, \bar{N}_3]$ for a total population of $N_{\text{tot}}$ ion channels, and $W \in \mathbb{R}^3$ is a standard 4-dimensional Brownian motion. Recall that $\vec{\pi}$ is the leading eigenvector of the graph Laplacian $L$ which means that $\bar{N}_i = N_{\text{tot}}\pi_i$ will change as a function of $\alpha$, but we suppress this notation for clarity. We note that the size of the fluctuations on each edge is reflected in the columns of $B_0$ and that this is the same matrix we consider in the 3-state model with timescale separation shown in Equation 8.

For the linear additive Gaussian noise process, we showed in [10] that the stationary variance of the observable state 3 decomposes into a sum of contributions from each edge as given in Equations S9 and S10.

## Derivation of the Variance: 3-State Process

Lyapunov's equation (Eq 46) is well known to hold not only for linear Gaussian processes (multivariate Ornstein-Uhlenbeck processes) but also for discrete population processes in which the rates of transitions are linear functions of the population at each node, i.e. first-order transition networks, such as those we consider here (see [8], [44] page 1, ¶5).

First assume there is a single random walker $X(t)$ moving on states $\{1, 2, 3\}$ with transition rate $\alpha_{ij}$ from state $i$ to state $j$. Then we have three probabilities,

$$p_1(t) = \Pr(X(t) = 1) \quad \text{(S43)}$$
$$p_2(t) = \Pr(X(t) = 2) \quad \text{(S44)}$$
$$p_3(t) = \Pr(X(t) = 3) \quad \text{(S45)}$$

with $p_1 + p_2 + p_3 \equiv 1$ at all times $t$. The evolution equations for these probabilities are

$$\frac{dp_1}{dt} = -p_1(\alpha_{12} + \alpha_{13}) + p_2\alpha_{21} + p_3\alpha_{31} \quad \text{(S46)}$$
$$\frac{dp_2}{dt} = p_1\alpha_{12} - p_2(\alpha_{21} + \alpha_{23}) + p_3\alpha_{32} \quad \text{(S47)}$$
$$\frac{dp_3}{dt} = p_1\alpha_{13} + p_2\alpha_{23} - p_3(\alpha_{31} + \alpha_{32}) \quad \text{(S48)}$$

Consider the matrix $Q = L^\intercal$

$$Q = \begin{pmatrix} -(\alpha_{12} + \alpha_{13}) & \alpha_{12} & \alpha_{13} \\ \alpha_{21} & -(\alpha_{21} + \alpha_{23}) & \alpha_{23} \\ \alpha_{31} & \alpha_{32} & -(\alpha_{31} + \alpha_{32}) \end{pmatrix} \quad \text{(S49)}$$

and write the probability as a (row) vector $\mathbf{p}(t) = (p_1(t), p_2(t), p_3(t))$. Then we have

$$\frac{d\mathbf{p}}{dt} = \mathbf{p}(t)Q. \quad \text{(S50)}$$

In general, $Q$ can be a function of time; nothing in the following derivation changes, even if $Q$ is a discontinuous function (as in a voltage clamp experiment with sudden changes in the command potential). To keep notation simple we write $Q$ in place of $Q(t)$.

To proceed, let $N_i(t)$ be the function that is 1 when $X(t) = i$ and zero otherwise, for $i \in \{1, 2, 3\}$. Then the average of the vector $\mathbf{N} = (N_1, N_2, N_3)$ can be found componentwise,

$$E[\mathbf{N}](t) = (p_1, p_2, p_3) = \mathbf{p}(t) \tag{S51}$$

and satisfies

$$\frac{d}{dt}E[\mathbf{N}] = E[\mathbf{N}]Q. \tag{S52}$$

If we instead consider $N_{\text{tot}}$ random walkers moving independently, we end up with the same equation, since the averages of sums equal the sums of the averages. For the variance, we again consider a single random walker. If we have $N_{\text{tot}}$ individuals moving independently, then the variances sum, but the case $N_{\text{tot}} = 1$ is easier to work with.

Let $\mathbf{X} = (X_1, X_2, X_3)$ denote the row vector of indicator functions. Let $M = E[\mathbf{X}^\intercal\mathbf{X}]$ be the matrix of second moments, so $M_{ij} = E[X_iX_j]$, and recall the definition of the covariance

$$C = M - E[\mathbf{X}^\intercal]E[\mathbf{X}], \text{ that is, } C_{ij} = E[X_iX_j] - E[X_i]E[X_j]. \tag{S53}$$

Since there is only one particle, $X_iX_j \equiv 0$ whenever $i \neq j$. If $i = j$, then $X_iX_j = X_i^2 = X_i$ since $X_i$ is either zero or one. Therefore,

$$M = \text{diag}(E[\mathbf{X}]) = \text{diag}(\mathbf{p}), \tag{S54}$$

where diag($\mathbf{p}$) is the diagonal matrix with entries given by the components of the vector $\mathbf{p}$.

We wish to derive an expression for the rate of change of the covariance matrix $C(t)$, i.e. the instantaneous covariance of $\mathbf{X}$. (This is different from the lagged covariance matrix under stationary conditions).

Differentiating (S53),

$$\frac{d}{dt}C = \frac{d}{dt}M - \left(\frac{d}{dt}E[\mathbf{X}^\intercal]\right)E[\mathbf{X}] - E[\mathbf{X}^\intercal]\frac{d}{dt}E[\mathbf{X}] \tag{S55}$$

$$\frac{d}{dt}E[\mathbf{X}] = E[\mathbf{X}]Q \tag{S56}$$

$$\frac{d}{dt}E[\mathbf{X}^\intercal] = Q^\intercal E[\mathbf{X}^\intercal], \text{ so} \tag{S57}$$

$$\frac{d}{dt}C = \frac{d}{dt}M - Q^\intercal E[\mathbf{X}^\intercal]E[\mathbf{X}] - E[\mathbf{X}^\intercal]E[\mathbf{X}]Q. \tag{S58}$$

Since $M = \text{diag}(\mathbf{p})$, $dM/dt = \text{diag}(\mathbf{p}Q) = \text{diag}(\sum_i p_iQ_{i1}, \sum_i p_iQ_{i2}, \sum_i p_iQ_{i3})$ On the other hand, since $C = E[\mathbf{X}^\intercal\mathbf{X}] - E[\mathbf{X}^T]E[\mathbf{X}]$, we have $E[\mathbf{X}^\intercal]E[\mathbf{X}] = M - C$, so

$$\frac{dC}{dt} = \frac{dM}{dt} - Q^\intercal E[\mathbf{X}^\intercal\mathbf{X}] + Q^\intercal C - E[\mathbf{X}^\intercal X]Q + CQ.$$

Using the observation that

$$\text{diag}(\mathbf{p})Q = \begin{pmatrix} p_1 & & \\ & p_2 & \\ & & p_3 \end{pmatrix} \begin{pmatrix} q_{11} & q_{12} & q_{13} \\ q_{21} & q_{22} & q_{23} \\ q_{31} & q_{32} & q_{33} \end{pmatrix} = \begin{pmatrix} p_1q_{11} & p_1q_{12} & p_1q_{13} \\ p_2q_{21} & p_2q_{22} & p_2q_{23} \\ p_3q_{31} & p_3q_{32} & p_3q_{33} \end{pmatrix}$$

(or, in general $(\text{diag}(\mathbf{p})Q)_{ij} = p_iq_{ij}$), elementary algebra yields

$$\frac{dC}{dt} = \text{diag}(\mathbf{p}Q) - Q^\intercal\text{diag}(\mathbf{p}) - \text{diag}(\mathbf{p})Q + Q^\intercal C + CQ \tag{S59}$$

$$= Q^\intercal C + CQ + BB^\intercal \tag{S60}$$

where

$$BB^\intercal = \text{diag}(\mathbf{p}Q) - Q^\intercal\text{diag}(\mathbf{p}) - \text{diag}(\mathbf{p})Q \tag{S61}$$

$$= \begin{pmatrix} -2p_1q_{11} + \sum_i p_iq_{i1} & -p_1q_{12} - p_2q_{21} & -p_1q_{13} - p_3q_{31} \\ -p_1q_{12} - p_2q_{21} & -2p_2q_{22} + \sum_i p_iq_{i2} & -p_2q_{23} - p_3q_{32} \\ -p_1q_{13} - p_3q_{31} & -p_2q_{23} - p_3q_{32} & -2p_3q_{33} + \sum_i p_iq_{i3} \end{pmatrix}. \tag{S62}$$

But
$p_1q_{11}+p_2q_{21}+p_3q_{31}-2p_1q_{11} = p_2q_{21}+p_3q_{31}-p_1q_{11} = p_2q_{21}+p_3q_{31}-p_1(q_{12}-q_{13}) = p_2q_{21}+p_1q_{12}+p_3q_{31}+p_1q_{13}$. (For the 3-state chain model $q_{31} \equiv 0 \equiv q_{13}$, but here we allow for the general case). Similar arithmetic leads to

$$BB^\intercal = \begin{pmatrix} p_1q_{12}+p_2q_{21}+p_1q_{13}+p_3q_{31} & -p_1q_{12}-p_2q_{21} & -p_1q_{13}-p_3q_{31} \\ -p_1q_{12}-p_2q_{21} & p_1q_{12}+p_2q_{21}+p_2q_{23}+p_3q_{32} & -p_2q_{23}-p_3q_{32} \\ -p_1q_{13}-p_3q_{31} & -p_2q_{23}-p_3q_{32} & p_1q_{13}+p_3q_{31}+p_2q_{23}+p_3q_{32} \end{pmatrix}. \tag{S63}$$

Note this expression is valid whether or not we are in equilibrium. In equilibrium, if detailed balance holds, we would have $p_iq_{ij} = p_jq_{ji}$ for all $i$ and $j$, and the expression would simplify.

Now we see that it is always possible to find the matrix $B$ explicitly, i.e.

$$B = \begin{pmatrix} -\sqrt{p_1q_{12}} & \sqrt{p_2q_{21}} & -\sqrt{p_1q_{13}} & \sqrt{p_3q_{31}} & 0 & 0 \\ \sqrt{p_1q_{12}} & -\sqrt{p_2q_{21}} & 0 & 0 & -\sqrt{p_2q_{23}} & \sqrt{p_3q_{32}} \\ 0 & 0 & \sqrt{p_1q_{13}} & -\sqrt{p_3q_{31}} & \sqrt{p_2q_{23}} & -\sqrt{p_3q_{32}} \end{pmatrix}. \tag{S64}$$

Again, this expression is exact even out of equilibrium, when $\mathbf{p}$ is varying. (This also holds when $Q$ is a time varying function).

The steady state for $C$, when $Q$ is a constant matrix, therefore satisfies

$$CQ + Q^\intercal C = -BB^\intercal. \tag{S65}$$

In the stochastic shielding approximation, we replace $B$ with a new matrix

$$B_{\text{SS}} = \begin{pmatrix} -\epsilon_{12}\sqrt{p_1q_{12}} & \epsilon_{21}\sqrt{p_2q_{21}} & -\epsilon_{13}\sqrt{p_1q_{13}} & \epsilon_{31}\sqrt{p_3q_{31}} & 0 & 0 \\ \epsilon_{12}\sqrt{p_1q_{12}} & -\epsilon_{21}\sqrt{p_2q_{21}} & 0 & 0 & -\epsilon_{23}\sqrt{p_2q_{23}} & \epsilon_{32}\sqrt{p_3q_{32}} \\ 0 & 0 & \epsilon_{13}\sqrt{p_1q_{13}} & -\epsilon_{31}\sqrt{p_3q_{31}} & \epsilon_{23}\sqrt{p_2q_{23}} & -\epsilon_{32}\sqrt{p_3q_{32}} \end{pmatrix}, \tag{S66}$$

where the $\epsilon_{ij}$ reflect whether the noise contribution of edge $i \to j$ is included ($\epsilon_{ij} = 1$) or neglected ($\epsilon_{ij} = 0$).

We can now address the question of how to write the stationary variance (*e.g.* $C_{33}$, the variance of the open state) as a sum of contributions from the edges. As we previously observed (see the HH Na and K channel figures in [10]) under stationary conditions with detailed balance, the contribution of edges $i \to j$ and $j \to i$ are the same. This symmetry holds in the present case as well.

Suppose first that we set all the $\epsilon_{ij}$ to zero except for $\epsilon_{12}$ (call this edge, from 1 to 2, edge number 1, and the corresponding $\mathcal{B}$ matrix $\mathcal{B}_1$.). We would like to solve for the entire covariance matrix $C_1$ satisfying

$$C_1Q + Q^\intercal C_1 = -\mathcal{B}_1 = -B_1B_1^\intercal = \begin{pmatrix} -p_1q_{12} & p_1q_{12} & 0 \\ p_1q_{12} & -p_1q_{12} & 0 \\ 0 & 0 & 0 \end{pmatrix}. \tag{S67}$$

Since $C_1$ is symmetric we have to solve

$$\begin{pmatrix} \gamma_{11} & \gamma_{12} & \gamma_{13} \\ \gamma_{12} & \gamma_{22} & \gamma_{23} \\ \gamma_{13} & \gamma_{23} & \gamma_{33} \end{pmatrix} \begin{pmatrix} q_{11} & q_{12} & q_{13} \\ q_{21} & q_{22} & q_{23} \\ q_{31} & q_{32} & q_{33} \end{pmatrix} + \begin{pmatrix} q_{11} & q_{21} & q_{31} \\ q_{12} & q_{22} & q_{32} \\ q_{13} & q_{23} & q_{33} \end{pmatrix} \begin{pmatrix} \gamma_{11} & \gamma_{12} & \gamma_{13} \\ \gamma_{12} & \gamma_{22} & \gamma_{23} \\ \gamma_{13} & \gamma_{23} & \gamma_{33} \end{pmatrix} = p_1q_{12} \begin{pmatrix} -1 & 1 & 0 \\ 1 & -1 & 0 \\ 0 & 0 & 0 \end{pmatrix} \tag{S68}$$

where $\gamma_{ij} = \gamma_{ji}$ are the $(i, j)$ and $(j, i)$ entries of $C_1$. This is a system of six linear equations in six unknowns:

$$\gamma_{11}q_{11} + \gamma_{12}q_{21} + \gamma_{13}q_{31} = -\frac{1}{2}p_1q_{12} \tag{S69}$$

$$\gamma_{11}q_{12} + \gamma_{12}q_{11} + \gamma_{12}q_{22} + \gamma_{22}q_{21} + \gamma_{13}q_{32} + \gamma_{23}q_{31} = p_1q_{12} \tag{S70}$$

$$\gamma_{11}q_{13} + \gamma_{12}q_{23} + \gamma_{13}q_{33} + \gamma_{13}q_{11} + \gamma_{23}q_{21} + \gamma_{33}q_{31} = 0 \tag{S71}$$

$$\gamma_{12}q_{12} + \gamma_{22}q_{22} + \gamma_{23}q_{32} = -\frac{1}{2}p_1q_{12} \tag{S72}$$

$$\gamma_{13}q_{13} + \gamma_{23}q_{23} + \gamma_{33}q_{33} = 0 \tag{S73}$$

$$\gamma_{12}q_{13} + \gamma_{22}q_{23} + \gamma_{23}q_{33} + \gamma_{13}q_{12} + \gamma_{23}q_{22} + \gamma_{33}q_{32} = 0. \tag{S74}$$

The covariance matrix satisfies an equation of the form

$$M\vec{c} = \vec{b} \tag{S75}$$

where

$$\vec{c} = [\gamma_{11}, \gamma_{22}, \gamma_{33}, \gamma_{12}, \gamma_{23}, \gamma_{31}]^\intercal \tag{S76}$$

$$M = \left(\begin{array}{ccc|ccc} -2\alpha_{12} & 0 & 0 & \alpha_{12} & 0 & 0 \\ 0 & 2(-\alpha_{21}-\alpha_{23}) & 0 & \alpha_{21} & \alpha_{23} & 0 \\ 0 & 0 & -2\alpha_{32} & 0 & \alpha_{32} & 0 \\ \hline 2\alpha_{21} & 2\alpha_{12} & 0 & m_{44} & 0 & \alpha_{23} \\ 0 & 2\alpha_{32} & 2\alpha_{23} & 0 & m_{55} & \alpha_{21} \\ 0 & 0 & 0 & \alpha_{32} & \alpha_{12} & -\alpha_{12}-\alpha_{32} \end{array}\right) \tag{S77}$$

where $m_{44} = -\alpha_{12} - \alpha_{21} - \alpha_{23}$, $m_{55} = -\alpha_{21} - \alpha_{23} - \alpha_{32}$, and $\vec{b}$ is the right hand side determined by the entries of $BB^\intercal$. The $6 \times 6$ matrix $M$ has rank 5, so Eq S75 does not have a unique solution without an additional condition. For the full covariance matrix (with $N = 1$) we know that $\gamma_{ii} = p_i(1 - p_i)$ and $\gamma_{ij} = -p_i p_j$ for $i \neq j$. Consequently for this case we have the additional condition

$$\gamma_{11} + \gamma_{22} + \gamma_{33} + 2(\gamma_{12} + \gamma_{23} + \gamma_{31}) \equiv 0. \tag{S78}$$

The system of equations (S75) combined with (S78) has full rank.

Condition (S78) holds for any covariance matrix since the covariance of the sum $N_1 + N_2 + N_3$ is always zero (see proof in Eq S85 - S89). So, we can extend the linear system of equations to

$$\tilde{M}\vec{c} = \tilde{\vec{b}} \tag{S79}$$

where

$$\tilde{M} = \left(\begin{array}{ccc|ccc} -2\alpha_{12} & 0 & 0 & \alpha_{12} & 0 & 0 \\ 0 & 2(-\alpha_{21}-\alpha_{23}) & 0 & \alpha_{21} & \alpha_{23} & 0 \\ 0 & 0 & -2\alpha_{32} & 0 & \alpha_{32} & 0 \\ \hline 2\alpha_{21} & 2\alpha_{12} & 0 & m_{44} & 0 & \alpha_{23} \\ 0 & 2\alpha_{32} & 2\alpha_{23} & 0 & m_{55} & \alpha_{21} \\ 0 & 0 & 0 & \alpha_{32} & \alpha_{12} & -\alpha_{12}-\alpha_{32} \\ \hline 1 & 1 & 1 & 2 & 2 & 2 \end{array}\right) \tag{S80}$$

where $m_{44}$ and $m_{55}$ are defined in matrix $M$ (Eq S77), and $\tilde{\vec{b}}$ is a seven component vector: the first six components of which form $\vec{b}$, and the last component of which is zero.

Lastly, we know that $\tilde{M}\vec{c}_1 = \tilde{\vec{b}}_1$ has a unique solution, and $\tilde{M}\vec{c}_2 = \tilde{\vec{b}}_2$ has a unique solution, and $\tilde{M}\vec{c} = \tilde{\vec{b}}$ has a unique solution. Therefore, if $\vec{b} = \vec{b}_1 + \vec{b}_2$ then $\vec{c} = \vec{c}_1 + \vec{c}_2$. To see this, note that since $\vec{b} = \vec{b}_1 + \vec{b}_2$, it follows that $\tilde{\vec{b}} = \tilde{\vec{b}}_1 + \tilde{\vec{b}}_2$. Hence,

$$0 = \tilde{\vec{b}} - \tilde{\vec{b}}_1 - \tilde{\vec{b}}_2 \tag{S81}$$

$$= \tilde{M}\left(\tilde{\vec{b}} - \tilde{\vec{b}}_1 - \tilde{\vec{b}}_2\right) \tag{S82}$$

$$= \vec{c} - \vec{c}_1 - \vec{c}_2. \tag{S83}$$

This calculation confirms the details in Lemma 1 (see Results), and illustrates our application of the Fredholm alternative.

### Demonstration that the sum condition holds

Let $C$ be a covariance matrix, *i.e.* for some vector valued random variable $[X_1, \ldots, X_n]^\intercal$,

$$C_{ij} = E[(X_i - E[X_i])(X_j - E[X_j])].$$

Define $\Delta_i = X_i - E[X_i]$, so $C_{ij} = E[\Delta_i \Delta_j]$. Suppose the total $\sum_{i=1}^n X_i \equiv \text{const}$ is preserved. Equivalently, the vector $X$ is distributed such that, with probability 1, $\Delta_1 + \ldots + \Delta_n \equiv 0$. Then

$$\sum_{i=1}^n \sum_{j=1}^n C_{ij} \equiv 0 \tag{S84}$$

Proof:

$$\sum_{i=1}^n \sum_{j=1}^n C_{ij} = \sum_{i=1}^n \sum_{j=1}^n E[\Delta_i \Delta_j] \tag{S85}$$

$$= E\left[\sum_{i=1}^n \sum_{j=1}^n \Delta_i \Delta_j\right] \tag{S86}$$

$$= E\left[\sum_{i=1}^n \Delta_i \sum_{j=1}^n \Delta_j\right] \tag{S87}$$

$$= E\left[\left(\sum_{i=1}^n \Delta_i\right)^2\right] \tag{S88}$$

$$= 0. \tag{S89}$$

This condition holds for our networks because they conserve probability (or total population). And the sum includes each off-diagonal covariance term twice, hence the condition (S78) is equivalent.
